# Cdk5-mediated JIP1 phosphorylation regulates axonal outgrowth through Notch1 inhibition

**DOI:** 10.1186/s12915-022-01312-4

**Published:** 2022-05-17

**Authors:** Doo Soon Im, Alvin Joselin, Devon Svoboda, Tesuya Takano, Maxime W. C. Rousseaux, Steve Callaghan, Ruth S. Slack, Shin-ichi Hisanaga, Roger J. Davis, David S. Park, Dianbo Qu

**Affiliations:** 1 Department of Clinical Neurosciences, Hotchkiss Brain Institute, Cumming School of Medicine, University of Calgary, Calgary, AB T2N 4N1 Canada; 2 Department of Cellular and Molecular Medicine, University of Ottawa Brain and Mind Research Institute, University of Ottawa, Ottawa, ON K1H 8M5 Canada; 3 Department of Biological Sciences, Tokyo Metropolitan University, Hachioji, Tokyo 192-0397 Japan; 4 Program in Molecular Medicine, University of Massachusetts Medical School, Worcester, MA 01650 USA

**Keywords:** Axonal outgrowth, Cdk5, Itch, JIP1, Notch1, phosphorylation

## Abstract

**Background:**

Activated Cdk5 regulates a number of processes during nervous system formation, including neuronal differentiation, growth cone stabilization, and axonal growth. Cdk5 phosphorylates its downstream substrates located in axonal growth cones, where the highly expressed c-Jun N-terminal kinase (JNK)-interacting protein1 (JIP1) has been implicated as another important regulator of axonal growth. In addition, stringent control of the level of intracellular domain of Notch1 (Notch1-IC) plays a regulatory role in axonal outgrowth during neuronal differentiation. However, whether Cdk5-JIP1-Notch1 cooperate to regulate axonal outgrowth, and the mechanism of such joint contribution to this pathway, is presently unknown, and here we explore their potential interaction.

**Results:**

Our interactome screen identified JIP1 as an interactor of p35, a Cdk5 activator, and we sought to explore the relationship between Cdk5 and JIP1 on the regulation of axonal outgrowth. We demonstrate that JIP1 phosphorylated by Cdk5 at Thr205 enhances axonal outgrowth and a phosphomimic JIP1 rescues the axonal outgrowth defects in JIP1^−/−^ and p35^−/−^ neurons. Axonal outgrowth defects caused by the specific increase of Notch1 in JIP1^−/−^ neurons are rescued by Numb-mediated inhibition of Notch1. Finally, we demonstrate that Cdk5 phosphorylation of JIP1 further amplifies the phosphorylation status of yet another Cdk5 substrate E3-ubiquitin ligase Itch, resulting in increased Notch1 ubiquitination.

**Conclusions:**

Our findings identify a potentially critical signaling axis involving Cdk5-JIP1-Itch-Notch1, which plays an important role in the regulation of CNS development. Future investigation into the way this pathway integrates with additional pathways regulating axonal growth will further our knowledge of normal central nervous system development and pathological conditions.

**Supplementary Information:**

The online version contains supplementary material available at 10.1186/s12915-022-01312-4.

## Background

Cyclin-dependent kinase 5 (Cdk5) mainly functions in post-mitotic neurons due to the neuronal expression of its dominant regulatory partner, p35 [1–3]. Cdk5 deficiency in mice causes perinatal mortality with abnormal corticogenesis and cerebellar defoliation [4], and p35 deficiency leads to cortical lamination defects, seizure, and lethality in adult mice [5], suggesting an essential role of Cdk5 kinase activity in the central nervous system (CNS). Cdk5/p35 plays an essential role in regulating neuronal differentiation, growth cone stabilization, and axonal outgrowth by phosphorylating effectors [3, 6–9]. However, our understanding of the downstream effectors of Cdk5/p35 which regulate these developmental events is incomplete.

Previously identified and characterized as a scaffold protein, JNK interacting protein 1 (JIP1) plays an essential role in axonal development and neurite extension. JIP1 specifically localizes to the axonal growth cones and its deficiency impairs axonal elongation during neuronal differentiation [10]. Moreover, JIP1 links several pathways, which contributes to axonal development. JIP1 contributes to axonal development by means of its association with other proteins, such as AKT [11]. AKT and JIP1 offer reciprocal protection to each other against proteasomal degradation in the axonal growth cone resulting in the augmentation of axonal outgrowth [11]. Tyrosine phosphorylation of JIP1 by c-Abl at the growth cone promotes axonal development in cortical neurons [10]. Considering its crucial involvement in axonal development, other mechanisms of JIP1 regulation likely influence its role in this process.

A particularly important interacting partner of JIP1 in this regard is Notch1. Notch1 controls axonal outgrowth and guidance in postmitotic neurons [12–14] and upregulation of Notch1 leads to premature cessation of axon elongation in maturing neuronal cultures [14]. The intracellular domain of Notch1 (Notch1-IC) also plays an antagonistic role in neurite outgrowth [13, 15, 16], suggesting the importance of Notch1 or Notch1-IC levels in determining the fate of neurons. Notch1-IC within the neuron is regulated through several mechanisms [17, 18]. One such mechanism involves the E3 ubiquitin ligase Itch which binds to Notch1 intracellular domain to promote its ubiquitination [17] and degradation, leading to its reduced activity [17, 18]. In addition, phosphorylation of Itch at different sites leads to contrasting effects on its E3 ligase activity [19, 20], suggesting the potential control of Itch E3 ligase function by selective phosphorylation. However, the mechanism by which Notch1 regulates axogenesis still remains unclear. Interestingly, not unlike Cdk5 or JIP1, Notch1 is also present in axons and dendrites of neurons [14, 21]. Notably, Notch1 is a downstream effector of JIP1 since the recombination signal binding protein for immunoglobulin kappa J region (RBP-JK) can impede the association of JIP1 and Notch1 resulting in the suppression of Notch1 activity [22].

Since our interactome study revealed JIP1 as a potential binding partner of p35, we investigated whether a functional interaction exists between Cdk5 and JIP1. We hypothesized that Cdk5-mediated JIP1 phosphorylation regulates axonal outgrowth through Notch1 inhibition. Here, we demonstrate that JIP1 is a novel substrate of Cdk5 and Cdk5-mediated phosphorylation of JIP1 at Threonine 205 (T205) promotes axonal outgrowth. Consistent with this observation, exogenous expression of a phosphomimic JIP1 T205D rescues the defects on axonal growth caused by Cdk5/p35 and JIP1 deficiency. A noticeable increase in Notch1-IC levels in JIP1-deficient neurons and the rescue of axonal outgrowth phenotype in these neurons by Notch1 inhibitor Numb point to a role of this signaling in JIP1/Cdk5-mediated axonal development. Importantly, JIP1 T205 phosphorylation by Cdk5 is mechanistically related to the upregulation of Cdk5-mediated Itch phosphorylation at Threonine 222 (T222) which enhances the ubiquitination and proteasomal degradation of Notch1-IC. Our results present a compelling delineation for the involvement of a novel Cdk5-JIP1-Notch1 axis in the regulation of axonal outgrowth.

## Results

### JIP1 interacts with p35 in vitro and in vivo

**Fig. 1:**
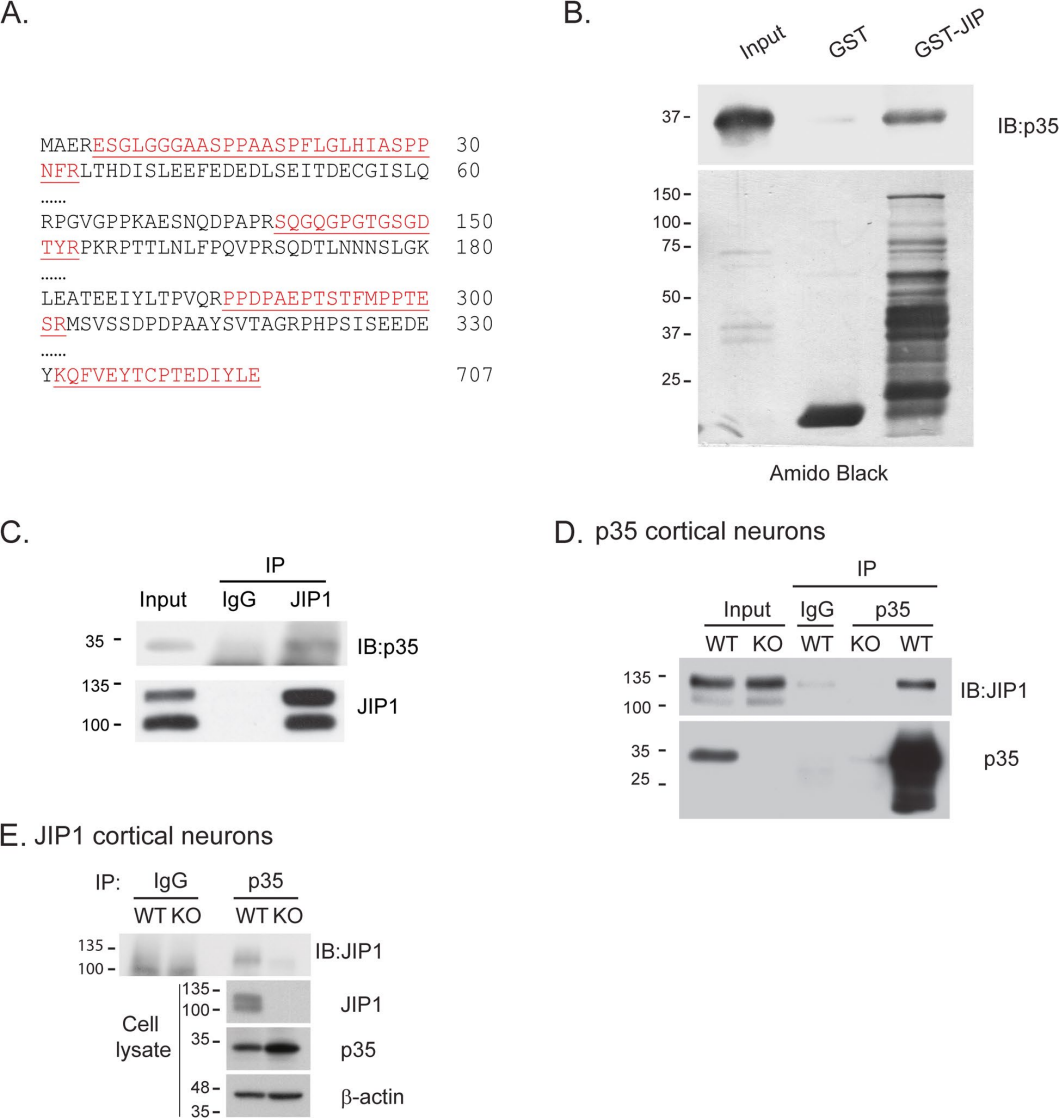
p35 interacts with JIP1. **A** Identification of JIP1 as p35-binding protein. Four peptides were identified and matched to mouse JIP1 by tandem mass spectrometry. **B** One microgram of bacterially expressed GST or GST fused-JIP1 was incubated with 2 μg His-p35 at 4°C for 2 h. The GST-tagged proteins were retrieved with GSH beads. The proteins were subjected to SDS-PAGE and detected using anti-p35 antibody by western blot. Amido black was used to visualize protein levels. **C** Endogenous p35 interacts with JIP1 in brain lysate. One microgram of IgG as control or JIP1 antibody was incubated with 500 μg of mouse brain lysate. Antibodies were pulled down using 50 μl goat-anti-rabbit IgG beads and the coupled proteins were subjected to SDS-PAGE followed by western blotting with anti-p35 antibody. **D** Confirmation of the specific interaction between p35 and JIP1 using p35 KO cortical neurons. Control IgG or anti-p35 was incubated with lysates from p35 WT or KO cortical neurons. The IPed proteins were immunoblotted with anti-JIP1 antibody. **E** Two-microgram control IgG or anti-p35 was incubated with lysate from JIP1 WT and KO neurons and the IPed proteins were immunoblotted with anti-JIP1 antibody. Images are representative of at least three independent experiments

Data from our previous mass spectrometry-based interactome screen [23, 24] identified four peptides that matched JIP1, indicating that JIP1 may interact with p35 (Fig. 1A). To further investigate whether Cdk5/p35 interacts with JIP1, an in vitro binding assay was performed using bacterially expressed GST, GST fused with JIP1, and His-tagged p35. p35 was only pulled down in the presence of GST-JIP1 but not with GST alone, suggesting a direct interaction between JIP1 and p35 (Fig. 1B). We further tested if this interaction could be observed endogenously in an immunoprecipitation (IP) assay using mouse brain lysates from embryonic (E) age of 15 to 16 days. Endogenous p35 co-immunoprecipitated with JIP1, but not with the IgG control (Fig. 1C). To confirm the specificity of the interaction, we performed a similar IP using extracts from cultured cortical neurons from p35 or JIP1 WT and KO mice. These experiments confirmed that the interaction of p35 and JIP1 was only observed in p35 WT (Fig. 1D) and JIP1 WT (Fig. 1E) neurons, but not in p35 KO, JIP1 KO neurons, or IgG controls. Taken together, these data suggest that JIP1 and p35 form a complex.

### Cdk5 phosphorylates JIP1 at Threonine 205

**Fig. 2:**
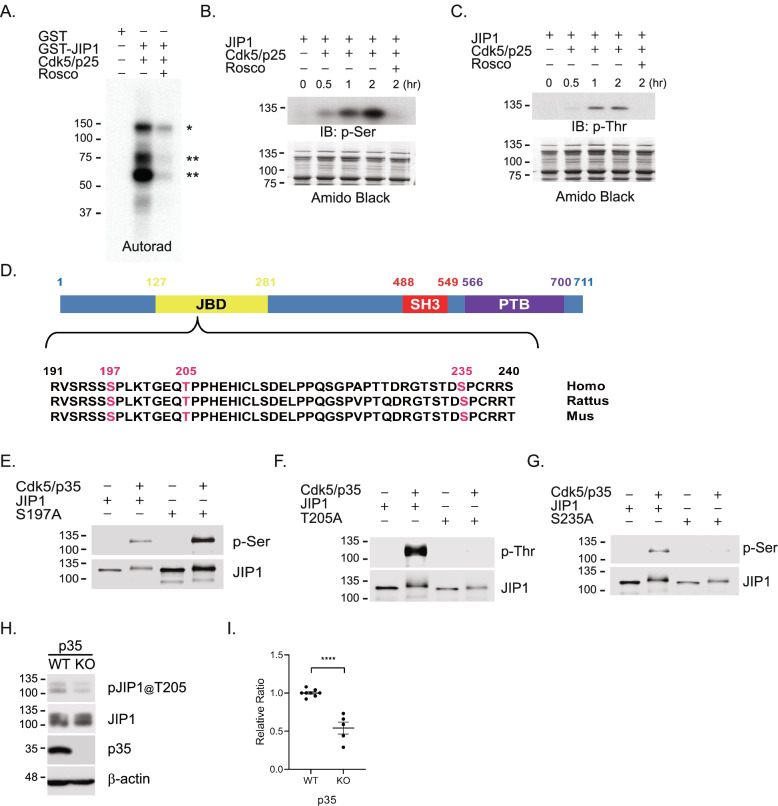
JIP1 is a substrate of Cdk5. **A** Bacterially expressed GST-tagged JIP1 was subjected to in vitro kinase assay by Cdk5/p25 and 1 μCi of [γ-^32^P] ATP with or without Cdk5 inhibitor Roscovitine (Rosco) at 30°C for 30 min. The proteins were separated by SDS-PAGE for autoradiography. **B**, **C** Recombinant His-JIP1 was incubated with Cdk5/p25 with or without 20 μM of Roscovitine for the indicated duration. The proteins were subjected to western blot with anti-phospho-Serine (p-Ser) (**B**) or anti-phospho-Threonine (p-Thr). * and ** indicate full length of JIP1 and truncated form of JIP1 from C-terminus, respectively (**C**). Data representative of three independent experiments. **D** Schematic of domains of JIP1. Alignment of JIP1 protein sequence from different species analyzed by ClustaIW2 to show three conserved sites optimal for Cdk5 phosphorylation. **E**–**G** Identification of Cdk5 phosphorylation sites in JIP1. Ser197, Thr 205, and Ser 235 were all replaced with Ala. An in vitro kinase assay was carried out with purified active Cdk5/p35 incubated with WT-JIP1 (JIP1) or its Ala mutants, S197A (**E**), T205A (**F**), and S235A (**G**) and examined by western blot analysis using anti-phospho-Serine (p-Ser) or anti-phospho-Threonine (p-Thr). Data representative of three independent experiments. **H** Total cell lysate from p35 WT and KO neurons at DIV 3 was subjected to western blot analysis with custom pJIP1@T205 antibody (bands at ~100 and ~120 kDa, pJIP1@T205). Both bands from p35 WT or KO neurons were used for the measurement of JIP1 phosphorylation by Cdk5. **I** Quantification of phospho-JIP1 T205 levels relative to p35 WT neurons. WT, *n*=8 and KO, *n*=5, “*n*” equals the number of animals. Student’s *t*-test was used for statistical analysis and the data are presented as mean ± SEM. **** *p* < 0.0001

The interaction of p35 and JIP1 indicates that JIP1 is a potential substrate of Cdk5. Since Cdk5 phosphorylates Proline (Pro or P)-directed Serine (Ser or S) or Threonine (Thr or S) in its substrates, we examined whether Cdk5 can phosphorylate JIP1 at these sites. We performed a radioactive in vitro kinase assay by co-incubating ^32^P-ATP with glutathione S-transferase (GST) or GST fused with JIP1 (GST-JIP1) with activated Cdk5, in the presence or absence of Cdk5 inhibitor, Roscovitine (Rosco). Phosphorylation of JIP1 was observed when JIP1 was incubated with activated Cdk5, which was notably decreased in the presence of Roscovitine (Fig. 2A). Further confirming this observation, serine phosphorylation (p-Ser) and Threonine phosphorylation (p-Thr) of JIP1 were observed in the presence of activated Cdk5 which further increased, in a time dependent manner, in a non-radioactive in vitro kinase assay. Both Ser and Thr phosphorylation of JIP1 was noticeably attenuated in the presence of Roscovitine (Fig. 2B, C). Based on these data, particularly from Fig. 2A, the ~60 kDa truncated GST-JIP1 which contains the N-terminal GST tag shows the most notable increase in phosphorylation. Taking into account the ~28 kDa molecular weight of GST, this fragment includes the first ~300 amino acidic residues at the N-terminus of JIP1. A specific search for preferred phosphorylation of Ser/Thr sites by Cdk5 within the consensus *motif* (S/T)PX (K/H/R) [3] that are also conserved across species in human, mouse, and rat resulted in the identification of 3 sites, S197, T205, and S235 at the N-terminus of JIP1 (Fig. 2D). To test if these sites are indeed the phosphorylation targets of Cdk5, wild-type JIP1 (WT-JIP1) and JIP1 non-phosphorylatable mutant constructs, with the respective Thr or Ser sites substituted with the non-phosphorylatable alanine (ala or A), S197A, T205A, and S235A, were subjected to an in vitro kinase assay. As shown in Fig. 2A, a pronounced decrease in Thr (Fig. 2F) and Ser (Fig. 2G) phosphorylation of JIP1 was observed when T205A or S235A was incubated with activated Cdk5 compared to WT-JIP1. However, no noticeable decrease in Ser phosphorylation was observed in S197A incubated with activated Cdk5 when compared to WT-JIP1 (Fig. 2E, lanes 2 and 4). These results suggest that T205 and S235 are potential Cdk5 phosphorylation sites on JIP1 and that S197 is not phosphorylated by Cdk5. To confirm if JIP1 is an endogenous substrate of Cdk5, we generated phospho-antibodies JIP1@T205 and JIP1@S235 against p-T205 and p-S235, respectively. The specificity of the phospho-antibodies to detect p-JIP1 was first tested in an in vitro kinase assay using recombinant proteins (Fig. S1A). We then tested if these phospho-antibodies indeed specifically detect p-JIP1 from whole cell extracts of cultured cortical neurons from JIP1 WT or KO mice by western blotting. Phospho-antibody JIP1@T205, but not JIP1@S235 (Fig. S1D) recognized bands corresponding to JIP1 in WT neurons (Fig. S1B) and was therefore used for all subsequent experiments. We also confirmed the specificity of JIP1@T205 in the human neuroblastoma cell line SH-SY5Y by overexpressing the abovementioned JIP1 T205A or a phosphomimic JIP1 T205D (Thr to Asp) mutant (Fig. S1C). Consistently, JIP1@T205 specifically recognized p-JIP1 in cells transfected with WT-JIP1 (JIP1) but not JIP1 T205A or JIP1 T205D. Finally, we tested the phosphorylation status of JIP1 in cultured cortical neurons from p35 KO mice. Distinct from human cells, two JIP1 bands (~100 and ~120 kDa) were detected by pJIP1@T205 and JIP1 antibody in JIP1 WT neurons, which correspond to the two known isoforms of JIP1. Both these bands were undetectable in JIP1 KO mouse (Fig. S1B). Both p-T205 levels compared to WT neurons and the relative ratio of p-T205 to total JIP1 were notably decreased in p35 KO mice (Fig. 2H, I). Taken together, our data indicate that T205 on JIP1 is a specific Cdk5 phosphorylation site and that the phospho-antibody JIP1@T205 specifically detects endogenous JIP1 phosphorylation at T205.

### JIP1 phosphorylated at T205 regulates axonal outgrowth in cultured cortical neurons

**Fig. 3:**
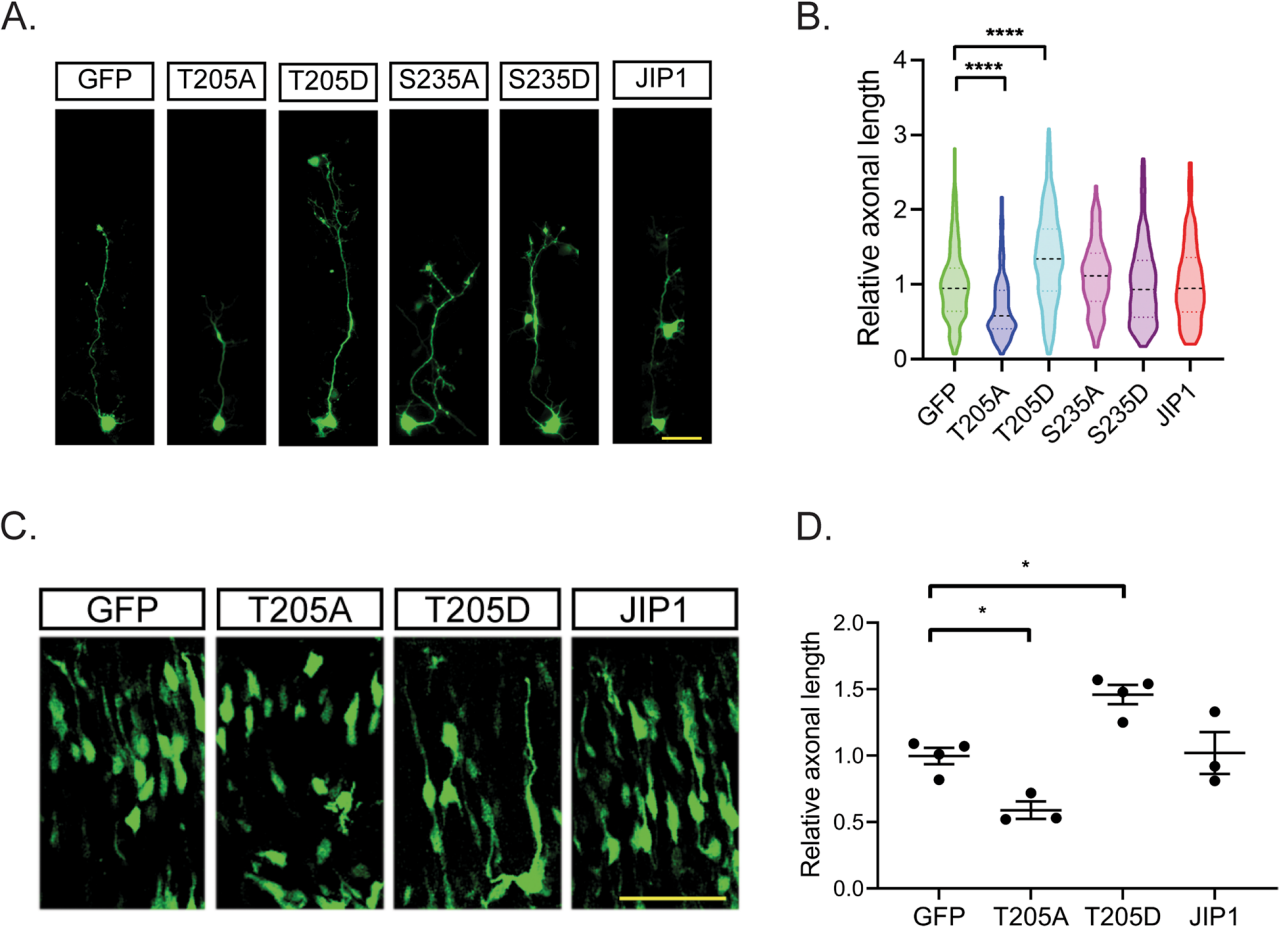
JIP1 Phosphorylated at Thr205 enhances axonal outgrowth in CD-1 cultured cortical neurons and in vivo. **A** Representative images at DIV 3 of CD-1 cortical neurons infected with adenoviral GFP control (GFP), WT-JIP1 (JIP1), its alanine mutants (T205A and S235A), or its aspartic acid mutants (T205D and S235D). Scale bar, 50 μm. **B** Quantification of axonal length relative to GFP control was analyzed by one-way ANOVA and are represented as mean ± SEM; *n*=227 for GFP, *n*=191 for T205A, *n*=179 for T205D, *n*=156 for S235A, *n*=171 for S235D, and *n*=154 for JIP1. “*n*” equals the number of neurons from one experiment. Data representative of 3 independent experiments. **C** Representative images of axonal outgrowth in CD-1 embryonic brains subjected to in utero electroporation at E 14.5 with either the GFP control or T205A, T205D, or JIP1 plasmid. All constructs express GFP under a separate promotor. Brains were collected for axonal length analysis 2 days after electroporation. **D** Quantification of axonal length relative to GFP control analyzed by one-way ANOVA. Data presented as mean ± SEM. 30 to 55 neurons per mouse, 4 mice each for GFP and T205D and 3 mice each for T205A and JIP1, were used for quantification. Scale bar, 50 μm. * *p* < 0.05 and **** *p* < 0.0001

To test whether T205 or S235 phosphorylated JIP1 regulates axonal outgrowth, CD-1 cortical neurons were infected with adenoviruses expressing GFP control (GFP), WT-JIP1, T205A, T205D, S235A, or S235D at the time of plating (days in vitro (DIV) 0). At DIV3, axonal length of GFP-positive neurons was measured. Axon lengths of neurons expressing T205A, but not S235A, were significantly shorter than those expressing control GFP. In contrast, expression of T205D significantly increased axon length (Fig. 3A, B). Following from this result that T205 phosphorylated JIP1 functionally induces axonal outgrowth in cultured cortical neurons, we further investigated if this was also the case during embryonic development in vivo. We accomplished this by in utero electroporation of either the control GFP plasmid or the various JIP1 plasmids T205A, T205D, or WT-JIP1 (JIP1) into the lateral ventricle of the embryonic brain at embryonic day (E) 14.5 to 15.5. Consistent with our in vitro data, 2 days after in utero electroporation, a significant reduction of axonal length by about 40% was observed in neurons expressing T205A compared to the GFP control. On the other hand, axonal length was increased by about 40% in cells expressing the phosphomimic T205D (Fig. 3C, D). No notable changes in axonal length were observed with either mutants of S235, suggesting that this site, although a Cdk5 in vitro phosphorylation site, likely does not play a role in the regulation of axonal outgrowth. To summarize, our in vitro and in vivo data together indicate that phosphorylation of JIP1 at T205 regulates axonal outgrowth during neuronal development.

### Phosphorylated JIP1 rescues the defects of axonal outgrowth in JIP1^−/−^ or p35^−/−^ neurons

**Fig. 4:**
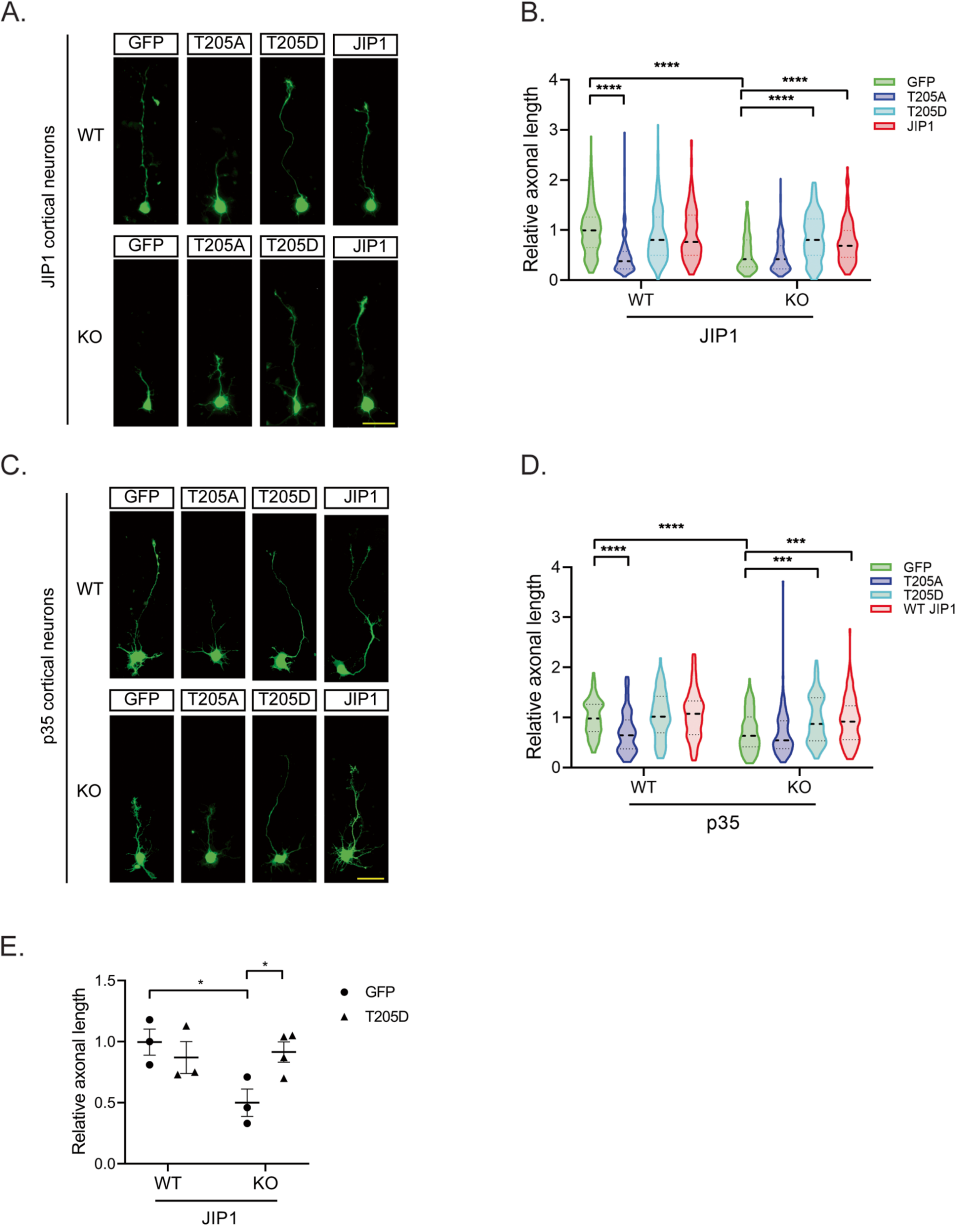
Phosphorylated JIP1 rescues the defect in axonal length in JIP1 KO and p35 KO neurons. **A** Representative images of JIP1 WT and KO neurons expressing GFP, T205A, T205D, and JIP1. Axon length of GFP positive neurons was analyzed 3 days after the infection. Scale bar, 50 μm. **B** Quantification of axon length relative to GFP control analyzed by two-way ANOVA. Data presented as mean ± SEM; *n*=233 for GFP, *n*=230 for T205A, *n*=231 for T205D, and *n*=198 for JIP1 for JIP1 WT neurons, and *n*=224 for GFP, *n*=227 for T205A, *n*=97 for T205D, and *n*=151 for JIP1 for JIP1 KO neurons. “*n*” represents the number of neurons from one experiment. **** indicates *p* < 0.0001. **C** Representative images of axonal length of p35 KO or WT neurons expressing GFP, T205A, T205D, and JIP1 3 days after infection. Scale bar, 50 μm. **D** Quantification of axonal length relative to GFP control analyzed by two-way ANOVA. Data presented as mean ± SEM; *n*=87 for GFP, *n*=85 for T205A, *n*=81 for T205D, and *n*=72 for JIP1 for p35 WT neurons, and *n*=138 for GFP, *n*=114 for T205A, *n*=72 for T205D, and *n*=116 for JIP1 for p35 KO neurons. “*n*” equals the number of neurons from one experiment and representative of 3 independent experiment. *** *p* < 0.001 and **** *p* < 0.0001. **E** Quantification of axon length in E14.5 embryonic brains subjected to in utero electroporation with GFP and T205D plasmids. Brains were collected and stained with anti-GFP for the measurement of axonal length 3 days after electroporation. Data presented as mean ± SEM. Student’s *t*-test was used to compare groups of data; *n*=3 for GFP and T205D for JIP1 WT brains, and *n*=3 for GFP and *n*=4 for T205D for JIP1 KO brains. “*n*” equals the numbers of animals and > 50 GFP-positive cells from each animal were used for quantification. * *p* < 0.05

Previous reports have demonstrated that loss of JIP1 or p35 leads to defect of axonal elongation during neuronal differentiation [9, 10]. Accordingly, we next tested the role of JIP1-T205 phosphorylation in the regulation of axonal defects in JIP1 and p35 deficient neurons. Cortical neurons at DIV0 from JIP1 or p35 WT and KO mice were infected with adenoviruses expressing GFP control, WT-JIP1, T205A, or T205D. At DIV3, axonal outgrowth was measured. In agreement with previous reports, we found that deletion of JIP1 or p35 in neurons significantly reduced axon length compared to WT neurons expressing control GFP reporter (Fig. 4A, C). Consistent with our above results, expression of T205A in neurons regardless of genotypes significantly inhibited axonal outgrowth compared to WT neurons expressing GFP as control. However, overexpression of either the phosphomimic T205D or JIP1 rescued axon length to those comparable to WT neurons (Fig. 4B, D). We then tested if these in vitro observations could also be translated to the regulation of axonal length in vivo. Either a GFP control or T205D construct was introduced into JIP1 WT or KO embryonic brain by in utero electroporation. Confirming our observations in vitro, axonal length deficiencies observed in JIP1 KO embryonic brains were almost entirely rescued by JIP1-T205D (Fig. 4E). Together, these results provide compelling evidence that phosphorylation of the JIP1 T205 has an important role in the regulation of axonal outgrowth.

### Increase of Notch1-IC regulates axonal outgrowth in JIP1^−/−^ cortical neurons

**Fig. 5:**
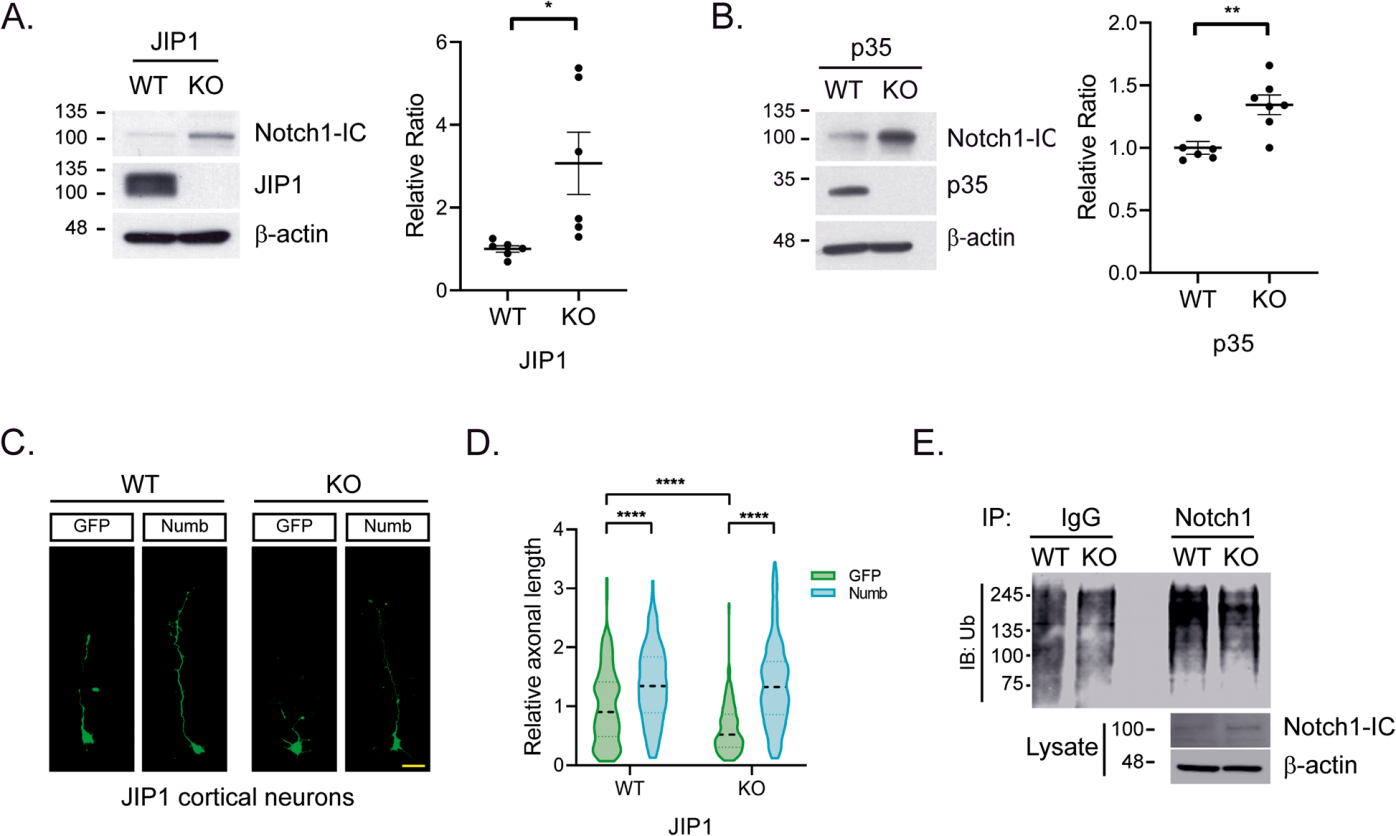
JIP1 deficiency regulates axonal outgrowth via Notch1 intracellular domain. **A** Western blot analysis of Notch1 in total cell lysate from JIP1 WT and KO neurons at DIV 3. Graph represents quantification of Notch1 intracellular domain (~100kDa, Notch1-IC) levels in JIP1 WT and KO (*n*=6 each). Data presented as mean ± SEM and analyzed by Student’s *t*-test. “n” equals the number of animals. * *p* < 0.05. **B** Total cell lysate from p35 WT and KO neurons at DIV 3 subjected to western blot analysis with anti-Notch1 antibody. Quantification of Notch1 intracellular domain (Notch1-IC) levels in p35 WT (*n*=6) and KO (*n*=7) is presented as mean ± SEM and analyzed by Student’s *t*-test. “*n*” equals the number of animals. ** *p* < 0.01. **C** Representative images of JIP1 WT and KO neurons infected with adenovirus carrying GFP or Numb. **D** Data was analyzed by two-way ANOVA and presented as mean ± SEM; *n*=163 for GFP, *n*=132 for Numb for JIP1 WT neurons and *n*=248 for GFP, *n*=247 for Numb for JIP1 KO neurons. “*n*” equals the number of neurons from one experiment. **** *p* < 0.0001. **E** JIP1 WT and KO neurons immunoprecipitated with Notch1 antibody followed by immunoblotting with ubiquitin (Ub) antibody

Given that Notch1 physically associates with JIP1 and its expression in cultured neurons leads to premature axon elongation [14], we explored if Notch1 might be involved in regulation of axonal growth mediated by Cdk5 phosphorylated JIP1. We first examined if Notch1-IC levels were affected in JIP1^−/−^ or p35^−/−^ neurons using western blot analysis. Notch1-IC levels in lysates from JIP1^−/−^ or p35^−/−^ neurons at DIV3 were significantly increased compared to extracts from the respective WT controls (Fig. 5A, B). Neither Notch2-IC nor Notch3-IC levels were similarly affected (Fig. S2A, B), suggesting a specific regulation of Notch1 activity by JIP1. Along these lines, we explored if a direct inhibition of Notch1 activity, bypassing JIP1 phosphorylation, might rescue some of the axon growth defects observed in JIP1 deficient neurons. We expressed Notch1 inhibitory protein, Numb, via adenoviral infection into cultured cortical neurons from JIP1^−/−^ mice. As anticipated, expression of Numb in JIP1^−/−^ neurons rescued the axonal length compared with GFP control neurons (GFP) (Fig. 5C, D). These data suggest a role for JIP1 in axonal growth regulation through inhibition of Notch1-IC level and therefore Notch1 activity.

### JIP1 T205 phosphorylation enhances Cdk5-mediated Itch phosphorylation and promotes Notch1 ubiquitination

**Fig. 6:**
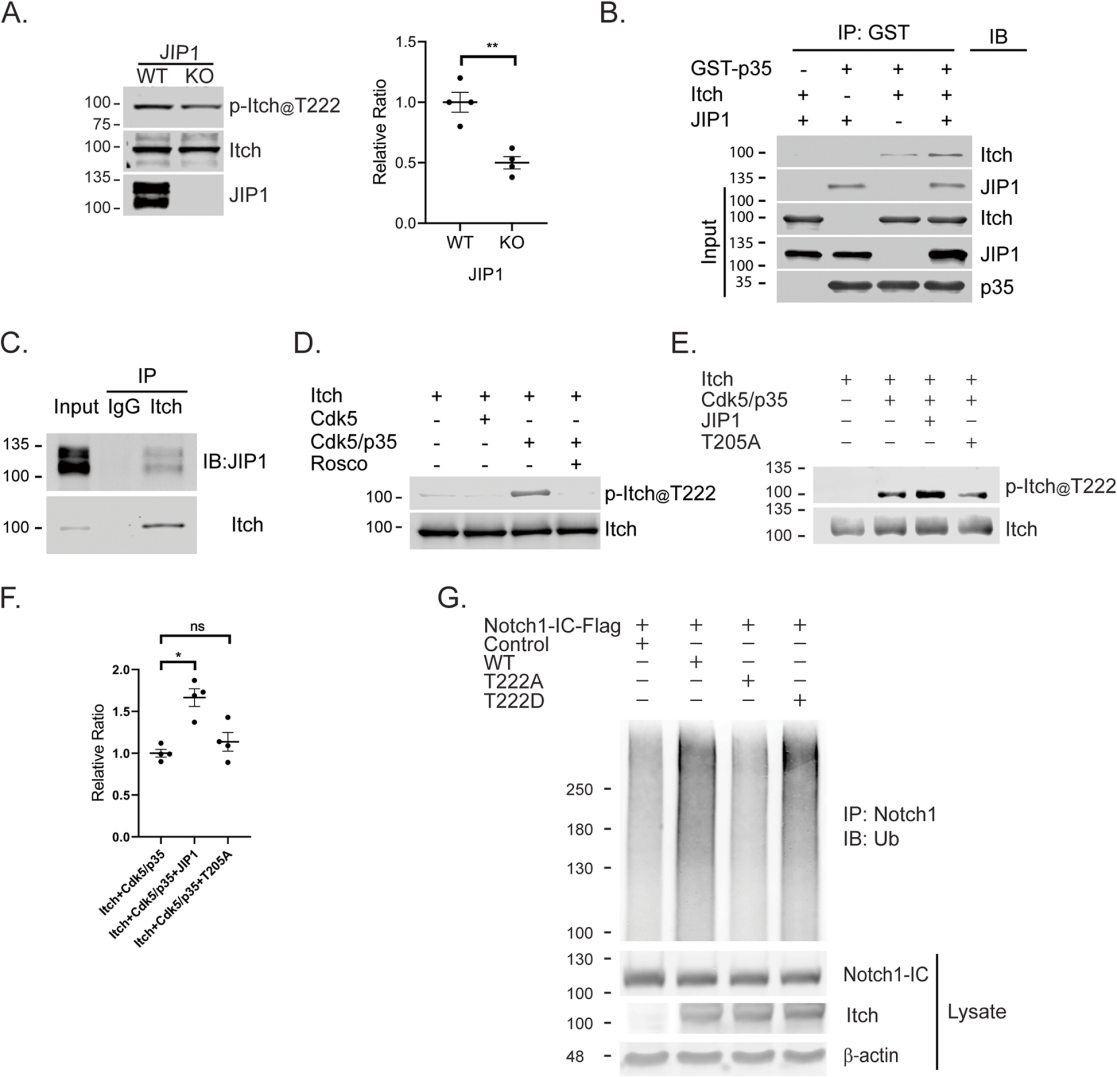
Phosphorylated JIP1 enhances Itch phosphorylation by Cdk5 to promote Notch1-IC ubiquitination. **A** Western blot analysis of phospho-Itch (p-Itch@T222) and Itch (Itch) in total cell lysate from JIP1 WT and KO neurons (DIV 3). Graph represents quantification of phospho-Itch levels relative to Itch in JIP1 WT and KO (*n*=4 each). Groups were compared using Student’s *t*-test and data presented as mean ± SEM. “*n*” equals the number of animals. ** *p* < 0.01. **B** Western blot analysis of in vitro binding assay using bacterially expressed GST or GST fused-p35 incubated with His-JIP1 and/or Itch recombinant proteins at 4 °C for 2 h. GST-tagged proteins were retrieved with GSH beads. The proteins were immunoblotted with Itch and JIP1 antibody. Data representative of three independent experiments. **C** Immunoblot analysis of CD-1 embryonic cortex lysate (E15.5-16.5) immunoprecipitated with Itch antibody and probed with JIP1 antibody. Data representative of three independent experiments. **D** Western blot analysis of Itch phosphorylation by Cdk5. Recombinant Itch protein incubated with Cdk5/p35 with or without 20 μM of Roscovitine (Rosco) at 30 °C for 2 h was immunoblotted with phospho-Itch (pItch@T222) and Itch antibody. Data representative of three independent experiments. **E** Western blot analysis of the contribution of JIP phosphorylation to Itch phosphorylation. Recombinant JIP1 or T205A was incubated with Cdk5/p35 at 30 °C for 1 h followed by the addition of Itch and incubated for 15 min. The mixture was then analyzed by immunoblotting with phospho-Itch and Itch antibody. **F** Quantification of phospho-Itch levels in E. Multiple comparison was analyzed by one-way ANOVA and data are presented as mean ± SEM from four independent experiments. * *p* < 0.05. **G** Wild-type Itch (WT) or Itch mutants (phosphomimic T222D (T222D) or Ala mutant T222A (T222A)) were co-transfected into HEK293A cells with Notch1-IC-Flag and immunoprecipitated with Notch1 antibody. Immunoprecipitated proteins were subjected to western blot analysis with Ubiquitin (UB) antibody. Data representative of three independent experiments

Since the interaction of E3 ligase Itch with Numb enhances the level of ubiquitinated Notch1 [17], we therefore examined whether JIP1 deficiency regulates Notch1 ubiquitination. Notch1 ubiquitination was reduced in JIP1 deficient neurons compared to WT controls (Fig. 5E). We next determined whether JIP1 deficiency affects levels of Numb and Itch since these proteins regulate Notch1 ubiquitination. JIP1 deficiency had no noticeable change in the expression level of Itch (Fig. S3A, B) or Numb (Fig. S3A, C). JNK phosphorylation of Itch at T222, which increases its E3 ligase activity, has been previously reported [20, 25, 26]. We therefore investigated if Notch1 ubiquitination and degradation could be regulated by phosphorylation of Itch by JNK or other kinases such as Cdk5 that in turn influence its E3 ligase activity. We first examined the levels of phosphorylated Itch and phosphorylated JNK with respect to their activities in JIP1-deficient neurons. Notwithstanding JIP1 deficiency, the level of phospho-JNK and phospho-c-Jun (p-c-Jun) remained unchanged compared to WT (Fig. S3D–F), suggesting that JNK may not be involved in the augmentation of Itch E3 ligase activity to regulate Notch1 degradation. By contrast, the level of phospho-Itch (Fig. 6A) was significantly reduced in JIP1^−/−^, indicating that other kinases likely regulate Itch E3 ligase activity. Since Itch has a canonical Cdk5 phosphorylation motif, TPRR, located at T222 optimal for Cdk5 phosphorylation, we first examined if Itch interacts with JIP1/Cdk5/p35 complex. As expected, Itch formed a complex with JIP1 and p35 in an in vitro binding assay (Fig. 6B). Consistently, the interaction of Itch and JIP1 was observed endogenously (Fig. 6C). We then tested if Cdk5 specifically phosphorylates Itch at T222 by an in vitro kinase assay. We observed a specific phosphorylation of Itch at T222 by Cdk5 which is abolished in the presence of Roscovitine (Rosco), indicating that Itch is a substrate of Cdk5 (Fig. 6D). Based on these data, we investigated whether p-JIP1 on T205 influences Cdk5-mediated Itch phosphorylation. We performed an in vitro kinase assay using purified recombinant protein JIP1, active Cdk5/p35 complex, and Itch. We found that co-incubation of Cdk5/p35 with JIP1 increased the level of phosphorylated Itch compared to Cdk5/p35 or co-incubation with T205A (Fig. 6E, F). This indicates that phosphorylated JIP1 on T205 regulates Itch phosphorylation by Cdk5. We next examined whether Itch phosphorylation had any effect on its binding with Notch1-IC. Itch T222D or T222A was co-transfected with Notch1-IC into HEK293 cells and an IP followed by western blotting performed after 48 h of expression. Increased Notch1 interaction was observed with T222D but not with T222A, suggesting that phosphorylated Itch has increased affinity for Notch1 interaction (Fig. S4). This assay lends further support to our hypothesis that Cdk5-mediated T222 phosphorylation of Itch impacts its interaction with its downstream substrate Notch1. Finally, we tested whether phosphorylated Itch could promote Notch1 degradation. Notch1-IC-FLAG was co-transfected into HEK293T cells with empty vector, Itch (WT), or Itch mutants, respectively. Twenty-four hours after transfection, the transfected cells were treated with 10 μM of MG132 for 4 h. As expected, overexpression of WT-Itch or T222D but not T222A increased Notch1 ubiquitination compared to the control group (Fig. 6G), suggesting that Cdk5-mediated Itch phosphorylation results in the increase of Notch1 ubiquitination.

## Discussion

Cdk5 is implicated in numerous aspects of CNS development and function, including neurite outgrowth [27]. Our present work aims to define Cdk5/p35 regulated effectors that contribute to its role in the shaping and regulation of CNS development. We demonstrate that p35 directly interacts with JIP1 and Cdk5 functionally phosphorylates only one of the three consensus phosphorylation motifs located at T205 in vitro and in vivo. Of the 2 sites that showed the most potential, expression of T205D, a phosphomimic mutant of JIP1, but not S235D mutant, functionally rescues the defects observed in JIP1 or p35 deficient neurons, suggesting a specific role for T205 phosphorylation in Cdk5-regulated axonal outgrowth.

JIP1 deficiency and p35 deficiency were previously reported to result in the dysregulation of neurite outgrowth [9, 10]. Here we present evidence that Cdk5-mediated phosphorylation of JIP1 at T205 plays an important role in regulating axonal outgrowth. This is particularly important since Cdk5/p35 in the embryonic brain plays a crucial role in regulating numerous key processes in CNS development. It should also be noted that expression of WT-JIP1 also rescued the defects in axon outgrowth in p35 deficient neurons. This is likely due to compensation by p39, another activator of Cdk5. In support of this observation, overexpression of p39 is known to stimulate neurite extension and this is blocked by overexpression of a dominant negative Cdk5 in immortalized hippocampal cells [28].

Previous studies have demonstrated that Notch family contributes to the regulation of neuronal development. For instance, overexpression of *Drosophila* Notch intracellular domain results in the loss of dorsal structures and expansion and brain disorganization [29]. In mammalian cells, Notch1 is most likely to play a role to switch the cell fate. Embryos carrying homozygous mutation in Notch1 die during gestation with widespread cell death [30], and activation of Notch1 is known to regulate dendritic branching positively and dendritic growth negatively [31]. Similarly, overexpression of Notch1 reduces axonal outgrowth in mammalian primary cortical neurons [14, 32]. JIP1 physically interacts with Notch1 and suppresses Notch1 transcriptional activity by the retention of Notch1 binding protein, RBP-Jk in the cytoplasm [22]. Our data shows that increased Notch1-IC level also results in an increase in Notch1 activity. Notch1 inhibitors, such as Numb, would be expected to mitigate the effect of increased Notch1 activity. Numb is a multi-functional protein [33], known to functionally inhibit Notch1 activity [17]. Along these lines, overexpression of Numb in JIP1^−/−^ neurons did rescue the deficits in axonal outgrowth. These observations strongly support a role of JIP1 in the regulation of Notch1 mediated axon outgrowth.

**Fig. 7:**
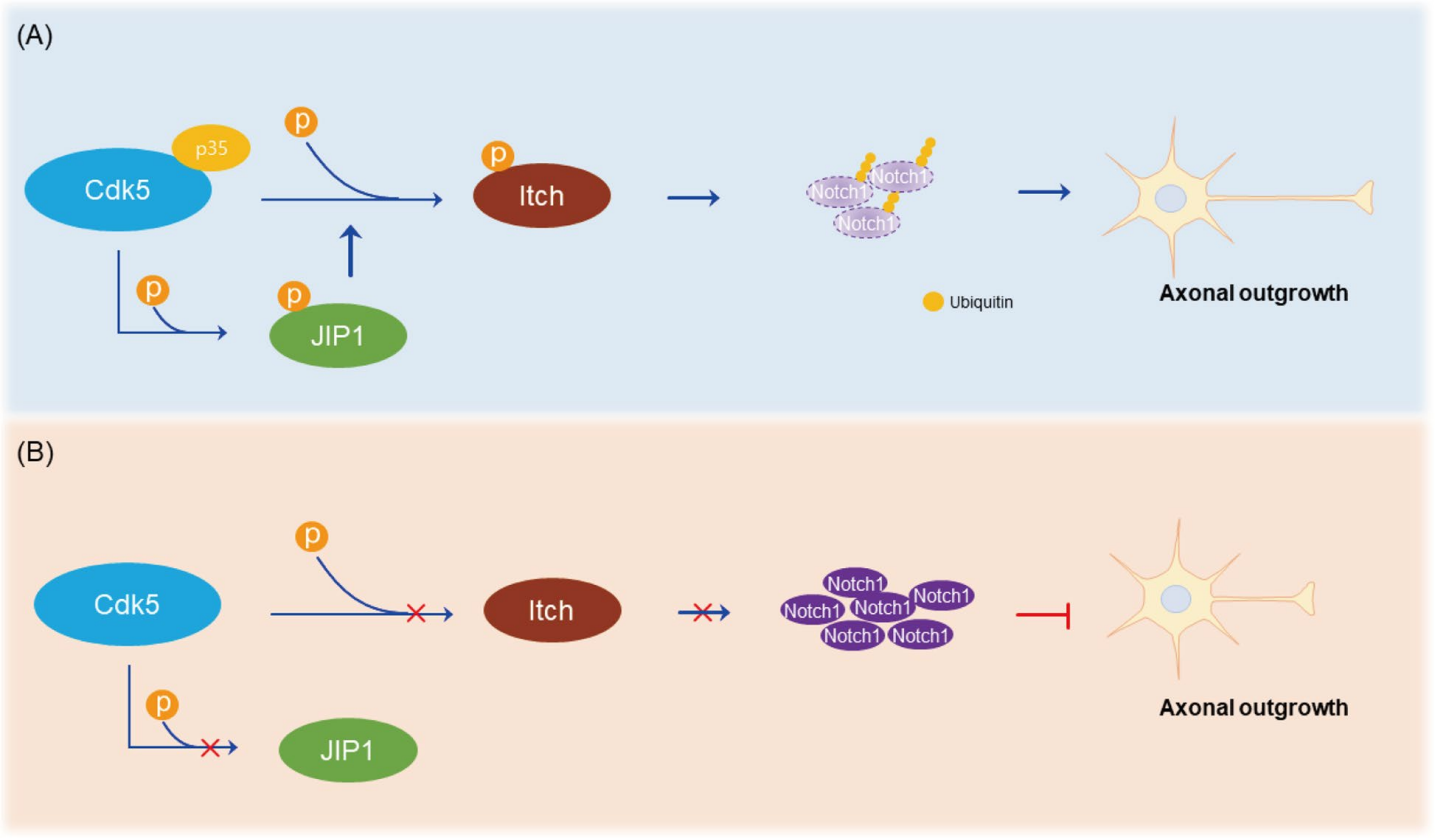
Illustration of Cdk5-JIP1-Notch1 axis in regulation of axonal outgrowth. **A** Cdk5/p35-mediated JIP1 phosphorylation boosts phosphorylation of Itch by Cdk5, which facilitates proteasome degradation of Notch1, resulting in enhanced axonal outgrowth. **B** Loss of JIP1 or p35 leads to an accumulation of Notch1, which result in inhibition of axonal outgrowth

E3 ligase Itch binds to Notch1-IC and increases Notch1 ubiquitination [34]. Phosphorylation of Itch at T222 by JNK plays a positive role in Itch-mediated proteasomal degradation [20]. We found that JIP1 deficiency leads to a decrease of ubiquitinated Notch1 and T222 phosphorylated itch levels in neurons but no change in Itch and Numb level, suggesting a role for Itch T222 phosphorylation in Notch1 degradation. It should be noted that the level of activated JNK is not affected by JIP1 deficiency in our study, meaning that kinases other than JNK likely regulate Itch phosphorylation. T222 on Itch is located within the canonical Cdk5 consensus phosphorylation motif, raising the probability that T222 is phosphorylated by Cdk5 and its E3 ligase activity is regulated by Cdk5-mediated phosphorylation. Increase of E3 ligase activity of Itch regulated by Cdk5 would facilitate Notch1-IC ubiquitination and degradation. Since phosphorylation of JIP1 by Cdk5 further promotes Cdk5-mediated Itch phosphorylation, the synergy of direct phosphorylation of Itch by Cdk5 and the amplification of Itch phosphorylation by phospho-JIP1 further escalates ubiquitination and degradation of Notch1-IC. Given that activation of Notch pathway leads to microtubules (MT) stabilization and inhibition of Notch pathway increases cytoskeletal plasticity [35], it is likely that rapid degradation of Notch is important in facilitating axonal elongation during neuronal development, particularly since the effect of Notch on microtubule-stabilization is a reversible processes that is finely regulated. Our data reveal that JIP1 phosphorylation by Cdk5 plays and essential role in the amplification of Itch phosphorylation by Cdk5 and loss of this loop is sufficient to impair axonal development (Fig.7).

Finally, we demonstrate that phosphorylation of JIP1 at T205 not only regulates axonal outgrowth in cultured neurons but can also facilitate axonal growth in mouse brain. It should be noted that since Cdk5^−/−^ or p35^−/−^ mice show severe defects in the positioning of postmitotic neurons during brain conformation [4, 36], we were unable to examine the effect of phosphorylated JIP1 on axonal outgrowth in these mice.

## Conclusions

A role for Cdk5/p35 in CNS development has been well documented in recent literature. The specific underlying pathways that this complex kinase is involved in, however, remain largely undiscovered. Our study presents strong evidence for the existence of a Cdk5/p35-JIP1-Notch1 axis that function together to regulate axon growth. Cdk5/p35 mediated JIP1 phosphorylation enhances Itch phosphorylation, which in turn promotes Notch1-IC degradation and axonal outgrowth. Importantly, Itch is itself a substrate of Cdk5/p35 and this phosphorylation is further enhanced by JIP1 phosphorylation, adding an additional level of regulation of this complex interaction. Further investigation of this novel axis may uncover additional pathways that will further extend our knowledge of this complex mechanism during CNS development and in other disease conditions.

## Methods

### Animals

CD1 mice were purchased from Charles River Laboratories. p35-deficient mice have been characterized by Hallows et al. [37]. Genotyping was performed as previously described [38]. JIP1-deficient mice that have been generated using methods described [39] were obtained from Dr. Roger J. Davis, and genotyped with primers, 5′-GTCCAGACTGCCTTGGGAAAA-3′, 5′-CGCGGTCTCAGGTGAGCAA-3′, and 5′-ATTGCTGCCTTCTGGAATGT-3′. All animal procedures were approved by the University of Ottawa Animal Care Committee and University of Calgary Animal Care Committee (ACC), and animals were maintained in strict accordance with the Guidelines for the Use and Treatment of Animals put forth by the Animal Care Council of Canada and endorsed by the Canadian Institutes of Health Research.

### Neuronal cell culture

Primary cortical neuronal culture was carried out as described previously [40]. Briefly, cortical neurons were dissected at embryonic days (E) 15.5–16.5 using CD1 (Charles River Laboratories, Code:022), JIP1, or p35 wild-type and knockout mice. Neurons were maintained in Neurobasal medium (Invitrogen, Cat. #21103-049) supplemented with B27 (Invitrogen, Cat. #17504-044), N2-supplement (Invitrogen, Cat. #17502-048), 0.5 mM L-glutamine (Gibco, Cat. #25030-081), and penicillin/streptomycin (Invitrogen, Cat. #15140-122) as previously described [41]. Primary cortical neurons with the density of 2.5 × 10^5^ or 7.0 × 10^6^ were plated into 24-well plate or 100-mm cell culture dish, separately. For viral infection, the cortical neurons were infected with adenovirus (MOI 30) at the time of plating. Neuronal cells were collected at the time points described in each figure legend.

### In utero electroporation

In utero electroporation was performed as previously described [42]. Briefly, Pups of pregnant females were electroporated at E14.5 with a plasmid containing JIP1 or its mutants with GFP under the control of a separate pCGI2 promoter. Plasmids were diluted to 2 μg/μl in sterile H_2_O and mixed with trace amounts of Trypan Blue dye. The plasmid was injected into the lateral ventricle of the brain using an Eppendorf Femtojet Microinjector and electroporated into the dorsolateral cortex using a BTX ECM 830 Square Wave Electroporator. Animals were sacrificed as described in figure legend. For analysis, electroporated sections at equivalent rostro-caudal levels of the brain, relative to the corpus callosum and anterior commissure, were selected.

### Antibodies

Mouse anti-JIP1 (B-7, Cat. #sc-25267, RRID: AB_627868), anti AIP4/Itch (G-11, Cat. #sc-28367, RRID: AB_667798), Rabbit anti-p35 (C64B10, 2680, C-19, Cat. #sc-820, RRID: AB_632137), Mouse anti-NUMB Antibody (48, Cat. #sc-136554, RRID: AB_10611794), Anti-c-Jun antibody (sc-74543, RRID: AB_1121646), and anti-Notch1 (mN1A, Cat. #sc-32745, RRID: AB_628033) were purchased from Santa Cruz Biotechnology (Texas, USA). Mouse anti-GFP (9F.F9, Cat. #ab1218, RRID: AB_298911), Anti-ITCH/AIP4 antibody (Cat. #ab220637), Rb pAb to Myc tag (Cat. #Ab9106, RRID: AB_307014), and Rb pAb to ubiquitin (Cat. #ab 19247, RRID: AB_444805) were purchased from abcam (Cambridge, UK). Rabbit anti-Notch1 (D1E11, Cat. #3608, RRID: AB_2153354), rabbit anti-Notch2 (D67CB, Cat. #4530P), rabbit anti-Notch3 (Cat. #2889S, RRID: AB_2298413), rabbit anti-Phospho-MAPK/CDK Substrates (PXS*P or S*PXR/K) (34B2, Cat. #2325S, RRID: AB_331820), monoclonal mouse anti-Phospho-Threonine-Proline (P-Thr-Pro-101) (Cat. #9391, RRID: AB_331801), SAPK/JNK antibody (Cat. #9252, RRID: AB_2250373), Phospho-SAPK/JNK (Thr183/Tyr185) antibody (Cat. #9251, RRID: AB_331659), Phospho-c-Jun (Cat. #3270s), and p35/25 (C64B10) rabbit mAB (Cat. #2680, RRID: AB_1078214) were purchased from Cell Signaling Technology (Massachusetts, USA). Mouse monoclonal anti-β-actin (Cat. #A5316, RRID: AB_476743) was purchased from Sigma-Aldrich (Missouri, USA). Itch polyclonal antibody (Cat. #PAS-72791, RRID: AB_2718645) was purchased from Invitrogen (California, USA). Rb p-Itch (Thr222) antibody (Cat. #AB 10050, RRID: AB_612038) was purchased from EMD Millipore (Massachusetts, USA). Special custom anti-phosphoJIP1 at Thr205 and anti-phosphoJIP1 at Ser235 were generated and purified from rabbit immunized with carrier protein-conjugated phosphopeptides, LKTGEQT^125^ (PO_3_) PPHEH, using standard protocols by PhosphoSolutions (Colorado, USA).

### Plasmid and recombinant protein preparation

Human JIP1 was amplified by RT-PCR using RNA isolated from SH-SY5Y as template. The amplified JIP1 was cloned in pAdtrack-CMV vector. Mouse Itch construct (Cat. # R210764) was purchased from Origene (Maryland, USA) and Itch was subcloned into pCI-neo with 3x-myc. FLAG-Notch1-IC, a gift from Nicholas Gaiano (Addgene plasmid # 26891; http://n2t.net/addgene:26891; RRID: Addgene_26891) [43]. JIP1 and Itch mutants were generated using Q5 site-directed mutagenesis kit from New England Biolabs (Massachusetts, USA) as described in manual. JIP1 and its mutants were subcloned into pCIG2 vector, pET28 for His-tagged recombinant proteins, and pGEX-4T-1 for GST-tagged recombinant proteins. To generate the constructs described above, primers indicated in Table S1 were used. All constructs were confirmed by DNA sequencing. Numb construct (MMM4769-202763993) was purchased from Thermo Fisher Scientific (Massachusetts, USA) and was cloned in pTrack-CMV-3xFlag vector. Cdk5, p35, and p35 were cloned in pGEX-4T-1 for bacterial expression of GST fusion protein and in pET-28a for His-tagged protein. Active Cdk5/p35 recombinant protein was purchased from Millipore Sigma (Cat. # 14-477). Recombinant Human ITCH/AIP4 isoform 2 protein was purchased from R&D system (Cat. # E3-260-100, Minnesota, USA).

### In vitro binding assay

The binding assay was performed as previously described [24]. Briefly, 1 μg of GST or GST-tagged JIP1 was incubated with 2 μg of His-p35 (Fig. 1) or GST-tagged p35 was incubated with 1 μg of Itch or/and 1 μg of His-JIP1 (Fig. 6). After 2-h incubation at 4 °C, the GST-tagged proteins were retrieved with GSH-Sepharose by further incubation for 1 h and then centrifugation. The beads were extensively washed and the bound proteins were subsequently released by boiling in SDS-PAGE sample buffer for 5 min. The released proteins were resolved by SDS-PAGE and detected by Western blotting.

### In vitro kinase assay

Cdk5 kinase assay was performed as previously described [24]. Briefly, for radioactive in vitro kinase assay, 2 μg of GST-JIP1 and 1 μCi [γ −32P]-ATP were incubated with Cdk5/p25 with or without Cdk5 inhibitor Roscovitine (Cat. #S1153, Selleck chemicals, TX, USA ) at 30 °C for 30 min. For non-radioactive in vitro kinase assay, 2 μg of His-JIP1 and mutants were incubated with 1 μg of GST-Cdk5/p35 with or without 20 μM of Roscovitine at 30 °C for indicated time points for Fig. 2. Recombinant Itch was incubated with active Cdk5/p35 with or without 20 μM of Roscovitine at 30 °C for 2 h or was incubated with active Cdk5/p35 in the presence of His-JIP1 or T205A at 30 °C for 1 h for Fig. 6. To stop the reaction, 2× SDS sample buffer was added to the tube, followed by boiling at 100 °C for 5 min. Western blot was performed to analyze phosphorylation.

### Western blot

Immunoblotting was performed as previously described [41]. Briefly, protein quantification was carried out using traditional Bradford method. Protein was separated with a 10% polyacrylamide gel in Tris-Glycine/SDS buffer and transferred onto polyvinylidene difluoride membrane (PVDF) for immunoblotting. Membranes were blocked in 5% non-fat dry milk except membranes which were used for detecting phospho-proteins which were blocked with 5% bovine serum albumin (BSA). Target proteins were probed with the following antibodies, mouse anti-JIP1 (diluted 1:1,000), rabbit anti-p35 (diluted 1:1000), customized phospho-JIP1 at Thr 205 (diluted 1:1000), customized phospho-JIP1 at Ser235 (diluted 1:1000), rabbit anti-Notch1 (diluted 1:500), rabbit anti-Notch2 (diluted 1:500), rabbit anti-Notch3 (diluted 1:500), rabbit anti-Itch (diluted 1:1000), rabbit anti-p-Itch (diluted 1:1000), mouse anti-itch (diluted 1:1000), mouse anti-Notch1 (diluted 1:1000), rabbit anti-ubiquitin (diluted 1:2000), goat anti-Itch (diluted 1:1000), rabbit anti p-SAPK/JNK (diluted 1:1000), rabbit anti SAPK/JNK (diluted 1:1000), mouse anti-Numb (diluted 1:1000), rabbit anti-Phospho-MAPK/CDK Substrates (diluted 1:1000), monoclonal mouse anti-Phospho-Threonine-Proline (diluted 1:1000), and Mouse anti-β-actin (1:40,000). After overnight incubation, membranes were washed with PBS. Membranes were incubated with the corresponding HRP conjugated secondary antibodies. Western blot signals were detected with ECL (Thermo Fisher, Cat. # 32106 or EMD Millipore, Cat. #WBKLS0500) or Odyssey Imaging Systems (LI-COR Biosciences, NE, USA). Quantification of western blots were performed by ImageJ densitometry (RRID: SCR_003070).

### Axonal length measurement

Immunofluorescent (IF) staining for brain tissue was performed as previously described [44]. Embryonic brains were collected at indicated time points after in utero electroporation (IUE). Brains were immersed in 4% paraformaldehyde for 1 day and incubated in 20% sucrose for 2 days at 4 °C. Sectioned brain at a thickness of 14 μm on a Microm cryostat microtome (Thermo Fisher Scientific, MA, USA) was permeabilized with 0.25% triton X-100 for 10 min, then incubated in 10% normal horse serum for 1 h for blocking. The blocked sections were incubated with mouse anti-GFP (diluted 1:400) overnight. Sections were thoroughly rinsed in PBS and incubated with Alex-488-conjugated anti-mouse IgG (1:200). All photomicrographs were taken using Zeiss LSM880 AxioObserver Z1 with AiryScan FAST (Oberkochen, Germany) or Zeiss Axoimager M2 (Oberkochen, Germany). Leading process of GFP-positive neuron on cortical plate was used for measurement of axonal length as described [6].

Axonal length from neuronal cell culture was measured at 3 days after plating and the GFP positive longest neurite of primary neurons was defined as the major axon as previously described [6]. The measurement of axonal length was carried out using Zeiss ZEN imaging software (Oberkochen, Germany).

### Immunoprecipitation (IP)

IP was carried out as previously described [41]. Briefly, samples were harvested in lysis buffer (25 mM Tris-HCl [pH 7.4], 150 mM NaCl, 1 mM EDTA, and 1 % Triton X-100) supplemented with protease inhibitors (Thermo Fisher Scientific, Cat. #A32963, Waltham, MA, USA). IPs were performed by incubating the antibodies with lysates overnight followed by incubation with Protein A or G beads (Sigma-Aldrich) at 4°C for 4 h. The beads were washed three times in lysis buffer without protease inhibitors. Western blot was performed to detect protein-protein interaction.

### Ubiquitination assay

Ubiquitination assay was performed as previously described [45]. Briefly, for Fig. 6G, 4.5 × 10^6^ HEK293T cells (ATCC, CRL-3216) in a 60-mm dish were transfected with 3 μg of FLAG-Notch1-IC in the presence of 6 μg of empty vector or Itch-Myc or Itch mutants utilizing 9 μl of Lipofectamine 2000. Twenty-four hours after transfection, the transfected cells were treated with 10 μM MG132 for 4 h. The total proteins were extracted in 100 μl of SDS sample buffer (50 mM Tris-HCl, pH6.8, 2% sodium dodecyl sulfate (*SDS*), 1% Glycerol). After 5 min of boiling and 20 min of centrifugation (20,000×*g*) at 4 °C, the supernatants were diluted 10 times with water for IP using 2 μg of Notch1 antibody and 30 μl of anti-mouse IgG beads (eBiosciences) for 2 h at 4 °C. The beads were washed four times using a wash buffer containing 20 mM Tris (pH 7.4), 300 mM NaCl, 0.1% SDS, and 1 mM EDTA. Proteins were eluted in 2× SDS sample buffer for separation by 4–15% SDS-PAGE gel (Bio-Rad) and Western blot analyses utilizing an anti-ubiquitin antibody.

For the ubiquitination assay of endogenous Notch1 using JIP1 WT and KO cortical neurons, cortical neurons at DIV 3 were harvested in lysis buffer and IPed with 2 μg of Notch 1 antibody. Western blot was performed with an anti-ubiquitin antibody. The assay was performed like that described above.

### Statistical analysis

All statistical analyses were employed by using GraphPad Prism software version 8.0 (RRID: SCR_002798). In this study, no blinding or randomization was performed. Data are expressed as the mean ± SEM. One-way or two-way analysis of variance (ANOVA) was used to compare multiple groups. Tukey test was used for post hoc comparisons after ANOVA. Unpaired two-tailed Student’s *t*-test was used to compare two groups.

## Supplementary Information

**Additional file 1: Table S1.** Primers for JIP1 and Itch constructs.

**Additional file 2: Fig. S1.** Specificity of anti- phospho-JIP1 antibody. **Fig. S2.** Notch2-IC or Notch3-IC level was unaffected by JIP1 deficiency. **Fig. S3.** Numb, Itch and phospho-JNK level were unchanged by JIP1 deficiency. **Fig. S4.** Phopho-Itch at Thr222 enhances interaction with Notch1-IC.

**Additional file 3.** Western blots.

**Additional file 4.** Individual data values.

## Data Availability

All data generated or analyzed during this study are included in this published article and its supplementary information files. All other data supporting the findings of this study are available from the corresponding author on reasonable request.

## Abbreviations

Cdk5: Cyclin-dependent kinase 5
JIP1: c-Jun N-terminal kinase (JNK)-interacting protein1
CNS: Central nervous system
Notch1-IC: Intracellular domain of Notch1
Notch2-IC: Intracellular domain of Notch2
Notch3-IC: Intracellular domain of Notch3
RBP-JK: Recombination signal binding protein for immunoglobulin kappa J region
DIV: Days in vitro
+/+: Wild-type
−/−: Knockout
IP: Immunoprecipitation
