# Supplementary material for: Cdk5-mediated JIP1 phosphorylation regulates axonal outgrowth through Notch1 inhibition
Source: BMC Biol. 2022 May 17;20:115. doi: 10.1186/s12915-022-01312-4 (PMC9115922; doi:10.1186/s12915-022-01312-4)
Supplement: Supplementary file 1 — Additional file 1: Table S1. Primers for JIP1 and Itch constructs. [file 12915_2022_1312_MOESM1_ESM.docx]

**Table S1. Primers for JIP1 and Itch constructs.**

| Table S1. Primers. F, Forward; R, Reverse | |
| --- | --- |
| Name | Sequence |
| JIP1 T205D F | 5’-CAGGGGAGCAGGATCCACCGCATGAACA-3’ |
| JIP1 T205D R | 5’-TGTTCATGCGGTGGATCCTGCTCCCCTG-3’ |
| JIP1 T205A F | 5’-CAGGGGAGCAGGCCCCACCGCATGAACA-3’ |
| JIP1 T205A R | 5’-TGTTCATGCGGTGGGGCCTGCTCCCCTG-3’ |
| JIP1 S235D F | 5’-ACCTCCACCGACGATCCTTGCCGCCGCA-3’ |
| JIP1 S235D R | 5’-TGCGGCGGCAAGGATCGTCGGTGGAGGT-3’ |
| JIP1 S235A F | 5’-ACCTCCACCGACGCCCCTTGCCGCCGCA-3’ |
| JIP1S235A R | 5’-TGCGGCGGCAAGGGGCGTCGGTGGAGGT-3’ |
| JIP1 S197A F | 5’-CTCGATCAATCCGCCCCCCTGAAGACAGG-3’ |
| JIP1 S197A R | 5’-CCTGTCTTCAGGGGGGCGGATGATCGAG-3’ |
| Itch T222D F | 5’-CCTTCGAGACCACCTCCACCCGATCCACGAAGACCAGCTTCTGTC-3’ |
| Itch T222D R | 5’-GACAGAAGCTGGTCTTCGTGGATCGGGTGGAGGTGGTCTCGAAGG-3’ |
| Itch T222A F | 5’-CCTTCGAGACCACCTCCACCCGCCCCACGAAGACCAGCTTCTGTC-3’ |
| Itch T222A R | 5’-GACAGAAGCTGGTCTTCGTGGGGCGGGTGGAGGTGGTCTCGAAGG-3’ |
