# Supplementary material for: Cdk5-mediated JIP1 phosphorylation regulates axonal outgrowth through Notch1 inhibition
Source: BMC Biol. 2022 May 17;20:115. doi: 10.1186/s12915-022-01312-4 (PMC9115922; doi:10.1186/s12915-022-01312-4)
Supplement: Supplementary file 2 — Additional file 2: Fig. S1. Specificity of anti- phospho-JIP1 antibody. Fig. S2. Notch2-IC or Notch3-IC level was unaffected by JIP1 deficiency. Fig. S3. Numb, Itch and phospho-JNK level were unchanged by JIP1 deficiency. Fig. S4. Phopho-Itch at Thr222 enhances interaction with Notch1-IC. [file 12915_2022_1312_MOESM2_ESM.docx]

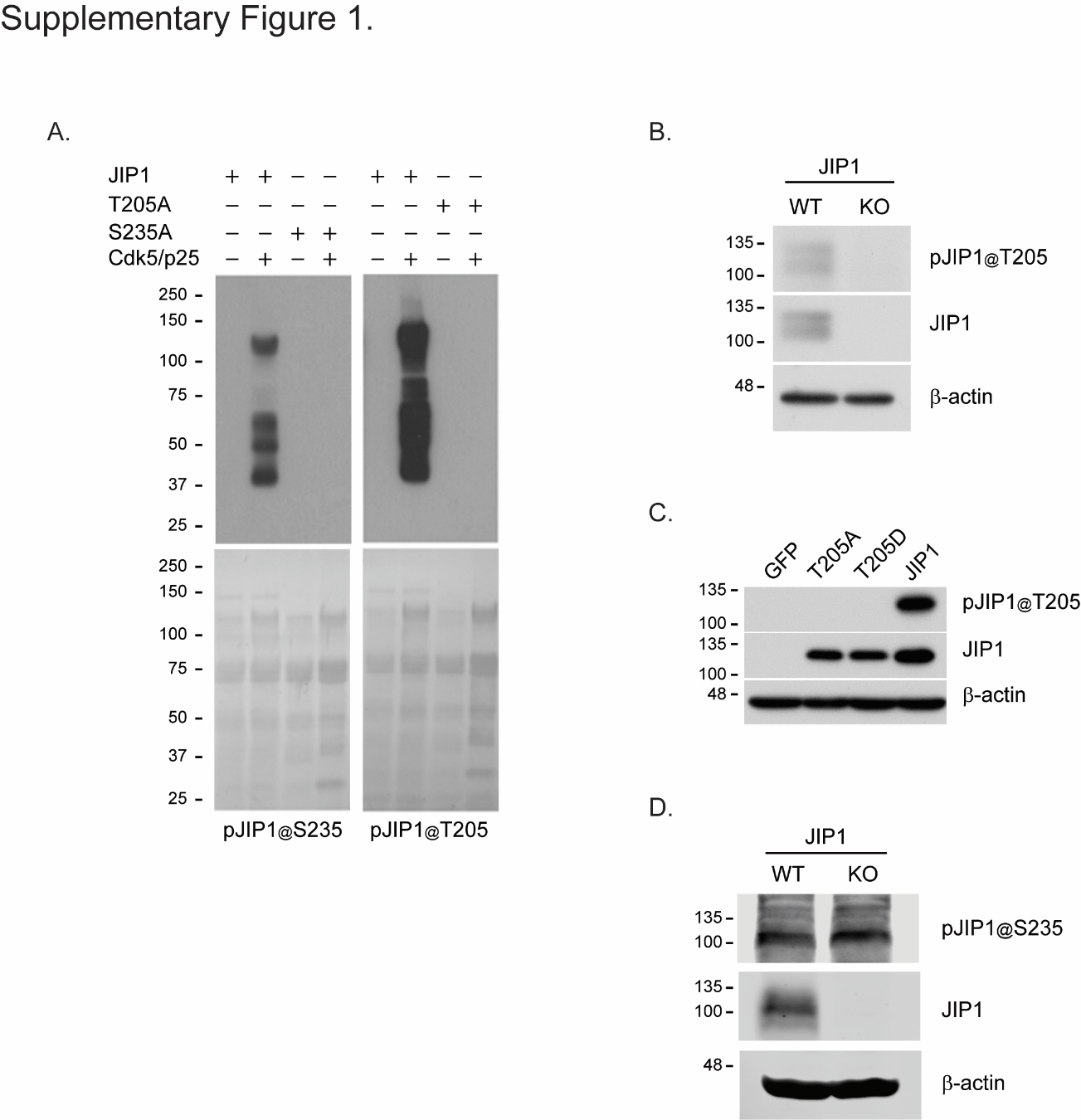


**Fig. S1. Specificity of anti- phospho-JIP1 antibody.** (A) Top panels: Recombinant JIP1 or its Ala mutants T205A and S235A was incubated with Cdk5/p35 at 30 °C for 2 h and was subjected to western blot using anti-phospho-JIP1 at S235 (pJIP1@S235) or T205 (pJIP1@T205). Bottom panels: Amido black staining. Experiment was replicated three times (B) Total cell lysate from JIP1 WT or KO neurons at DIV 7 was subjected to western blot analysis with custom generated pJIP1 T205 antibody. (Representative of three independent cell cultures). (C) Western blot analysis with custom phospho-JIP1 at T205 antibody of total cell lysate from SH-SY5Y cells transfected the plasmids, GFP, T205A, T205D or JIP1 for 48 h. (D) Cell lysate from JIP1 WT or KO embryonic brain was subjected to western blot analysis using anti-phospho-JIP1 at S235 (pJIP1@S235).


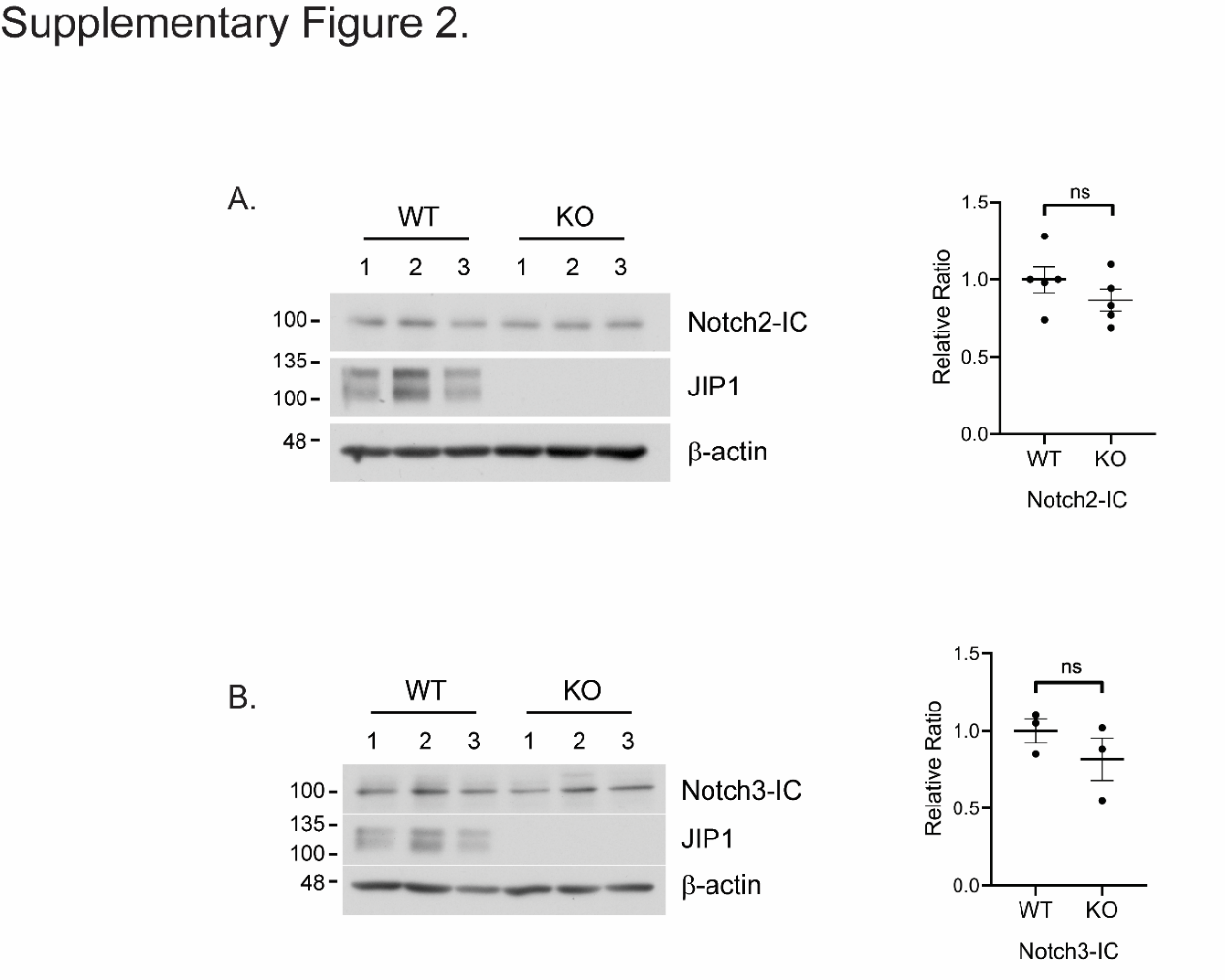


**Fig. S2. Notch2-IC or Notch3-IC level was unaffected by JIP1 deficiency.** Total cell lysates from JIP1 WT and KO neurons at DIV 3 were immunoblotted with Notch2 (A) or Notch3 (B) antibody. Notch2 intracellular domain (~100kDa, Notch2-IC, n=5 for WT, n=5 for KO) and Notch3 intracellular domain (~100kDa, Notch3-IC, n=3 for WT, n=3 for KO) levels in JIP1 WT and KO cortical neurons was quantified using ImageJ and analyzed by Student’s *t*-test. Data is presented as mean ± SEM. ‘n’ equals number of animals. ns; no significance.


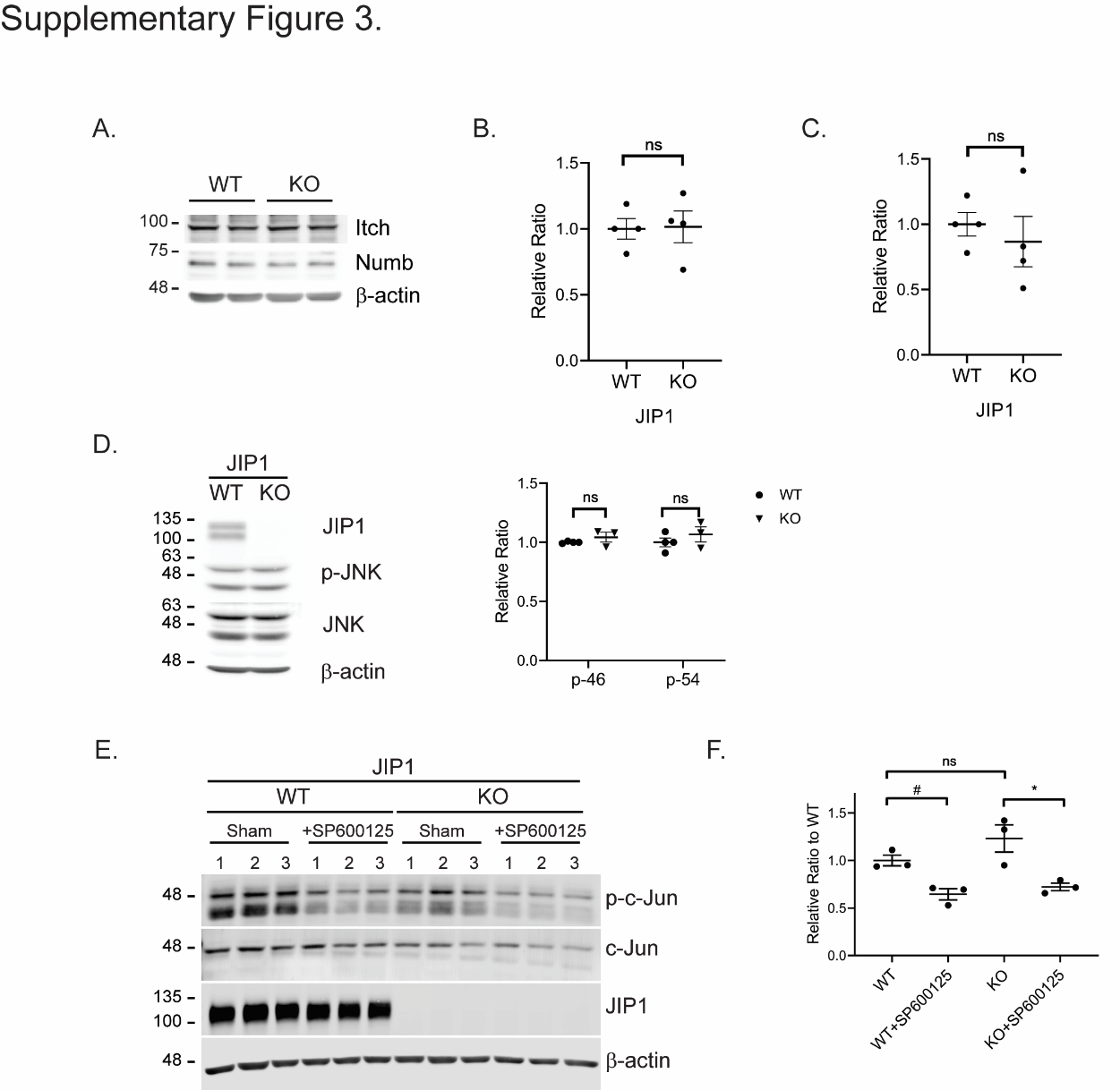


**Fig. S3. Numb, Itch and phospho-JNK level were unchanged by JIP1 deficiency.** Numb (n=4 for each group), Itch (n=4 for each group), phospho-JNK (p-JNK) or JNK (n=4 for WT, n=3 for KO group) level was analyzed in total cell from JIP1 WT and KO neurons at DIV 3. Column scatter plot represent quantification of Numb (B), Itch (C) and phospho-JNK relative to JNK (D). JIP1 WT and KO neurons treated with 20 μM of JNK inhibitor, SP600125, for 24h were collected at DIV3 and total cell lysates were used for western blotting with phospho-c-Jun (p-c-Jun) and c-Jun antibodies. Graph represents quantification of p-c-Jun levels in JIP1 WT and KO neurons with or without JNK inhibitor treatment (n=3 each group) (F). Data are expressed as relative ratio to WT and are presented as mean ± SEM and analyzed by one-way ANOVA, * *p* < 0.05 and Student’s *t*-test, # *p* < 0.05. ‘n’ equals the number of animals. ns; no significance.


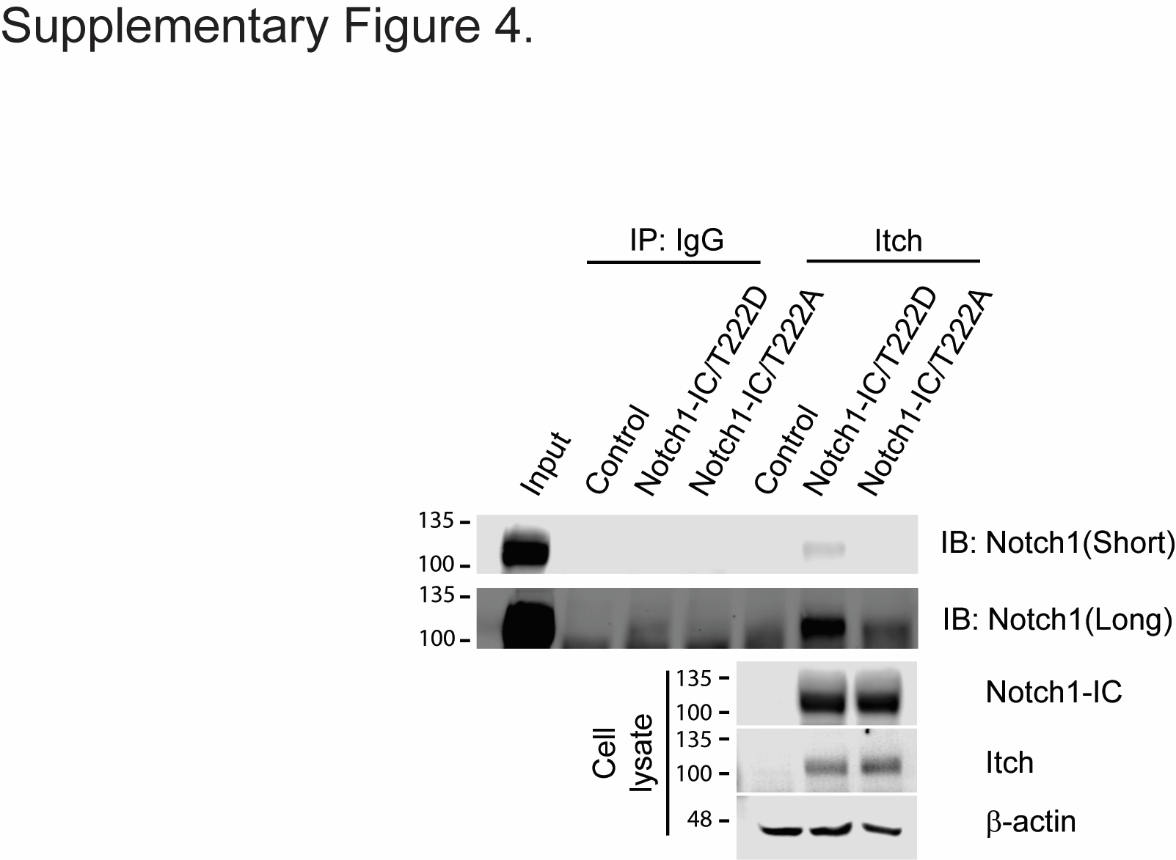


**Fig. S4. Phospho-Itch at Thr222 enhances interaction with Notch1-IC.** 4 μg of Itch mutant constructs, phosphomimic T222D (T222D), or Ala mutant T222A (T222A)) were co-transfected into HEK293A cells with Notch1-IC-Flag for 48 hr. Immunoprecipitation was carried out with Itch antibody. Immunoprecipitated proteins were subjected to western blot analysis with Notch1 antibody. Data is representative of two independent experiments.
