# Supplementary material for: Cdk5-mediated JIP1 phosphorylation regulates axonal outgrowth through Notch1 inhibition
Source: BMC Biol. 2022 May 17;20:115. doi: 10.1186/s12915-022-01312-4 (PMC9115922; doi:10.1186/s12915-022-01312-4)

Fig. 1B

Fig. 1C

Fig. 1D

Fig. 1E

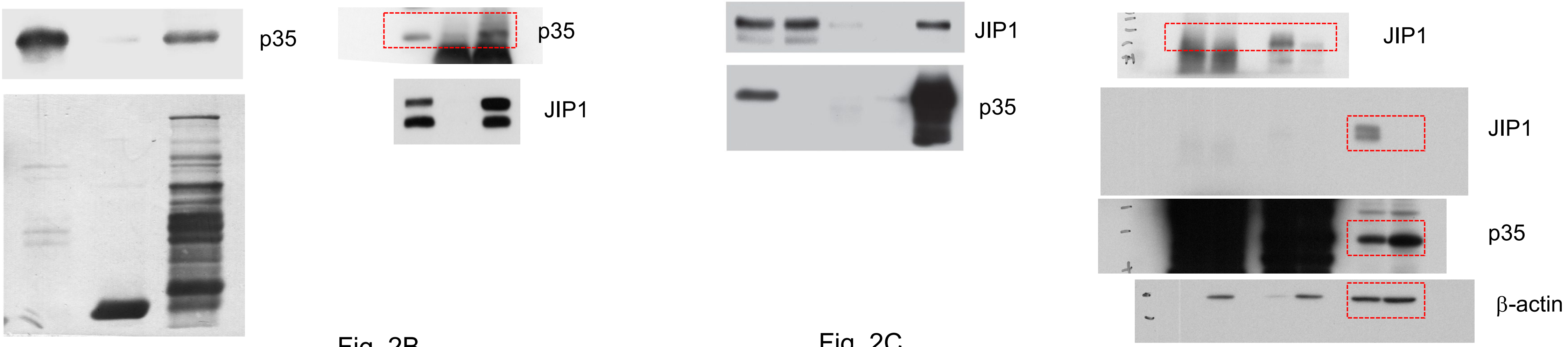

Fig. 2A

Fig. 2B

Fig. 2C

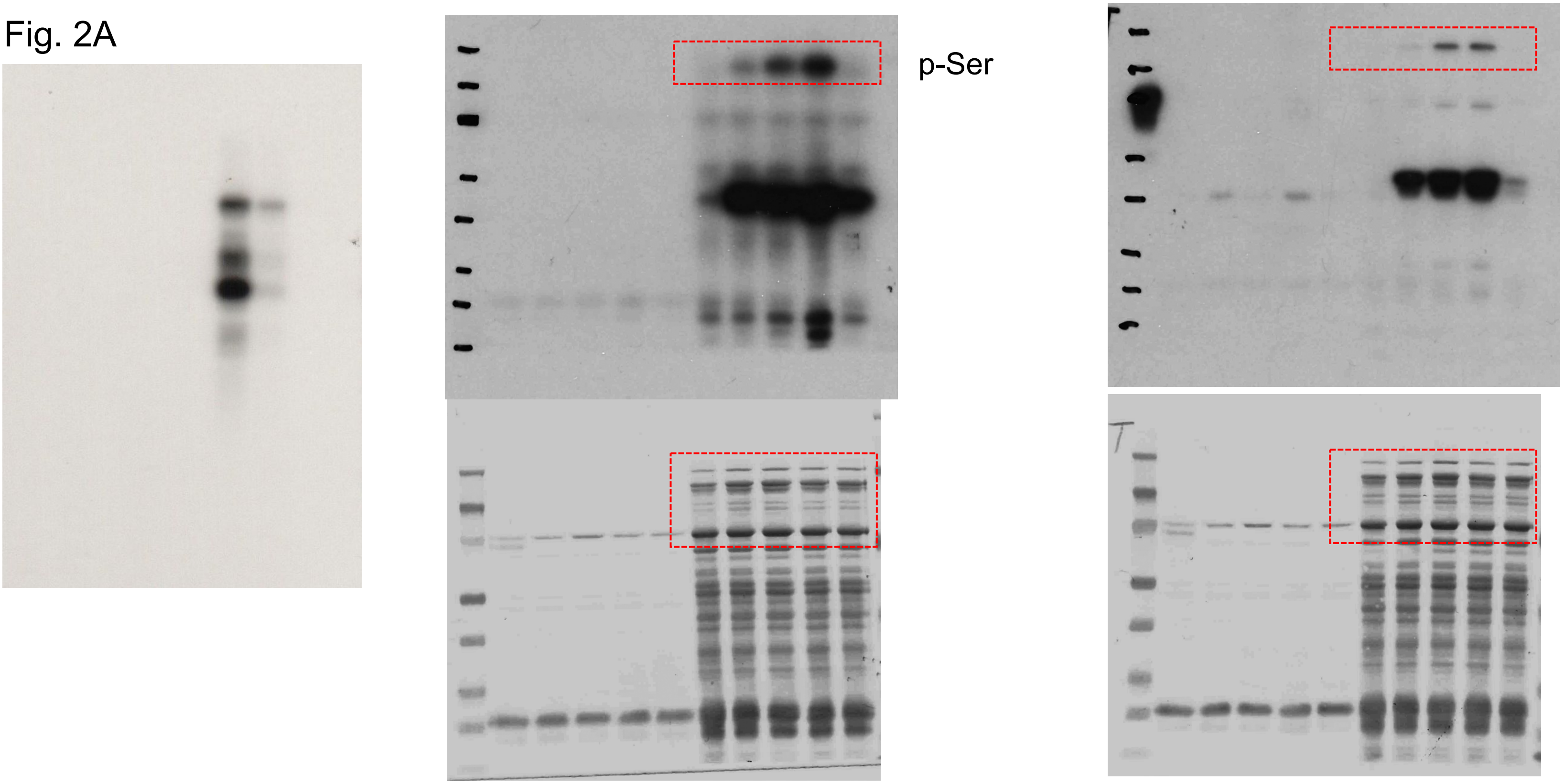

Fig. 2E

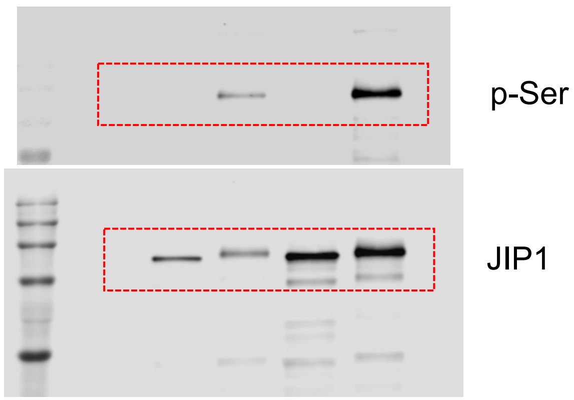

Fig. 2F

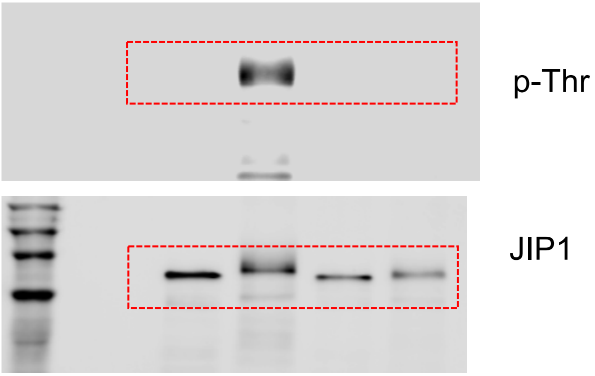

Fig. 2G

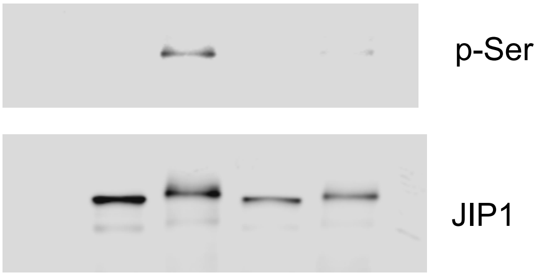

Fig. 2H

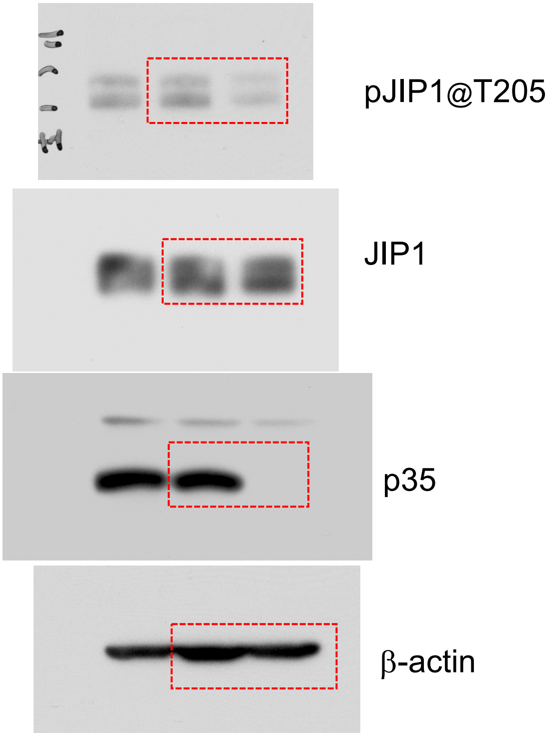

Fig. 5A

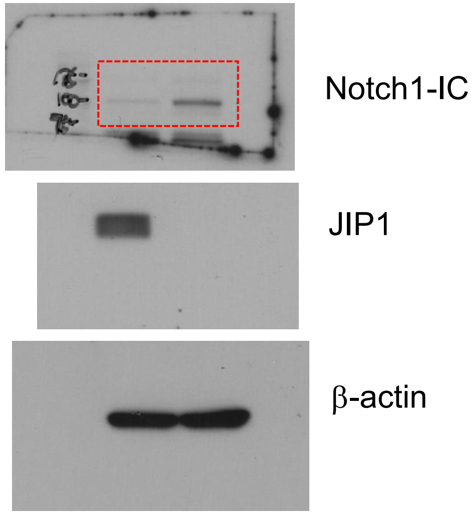

Fig. 5B

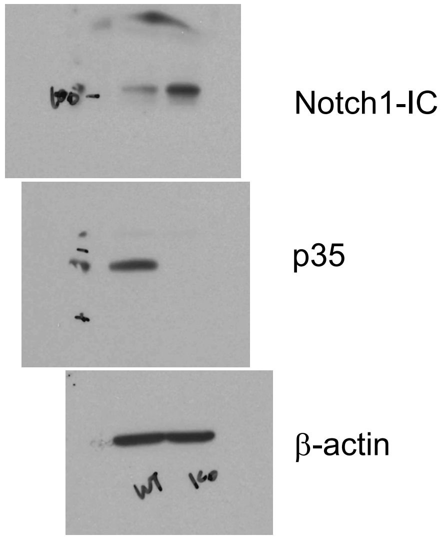

Fig. 5E

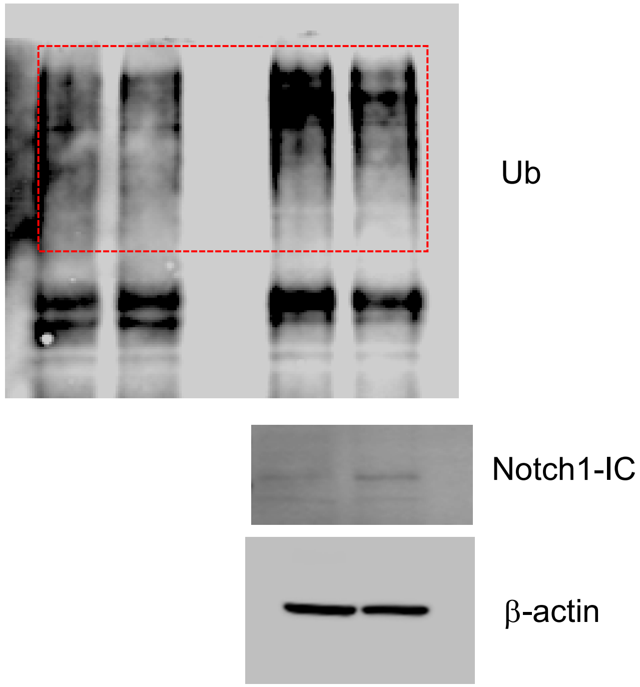

Fig. 6A

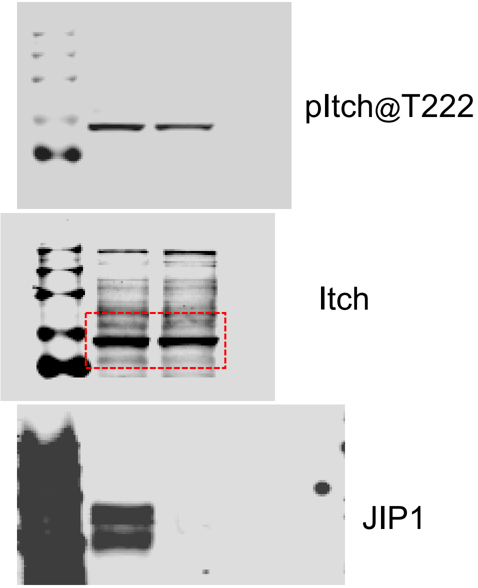

Fig. 6B

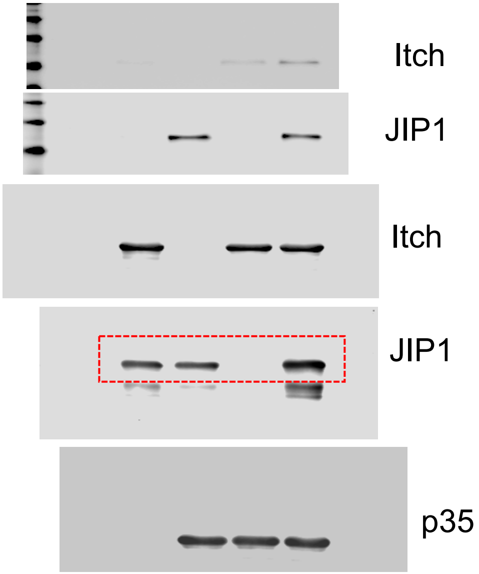

Fig. 6C

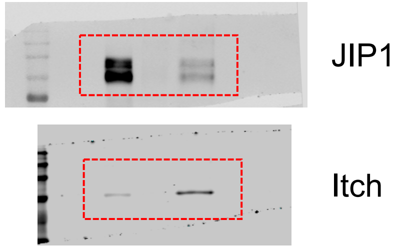

Fig. 6D

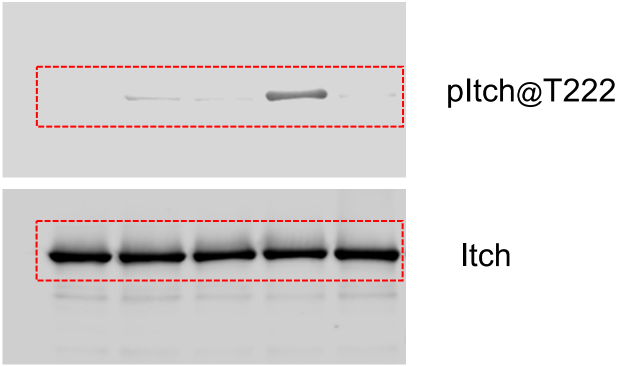

Fig. 6E

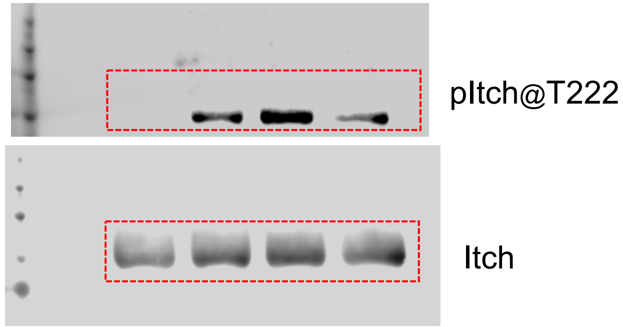

Fig. 6G

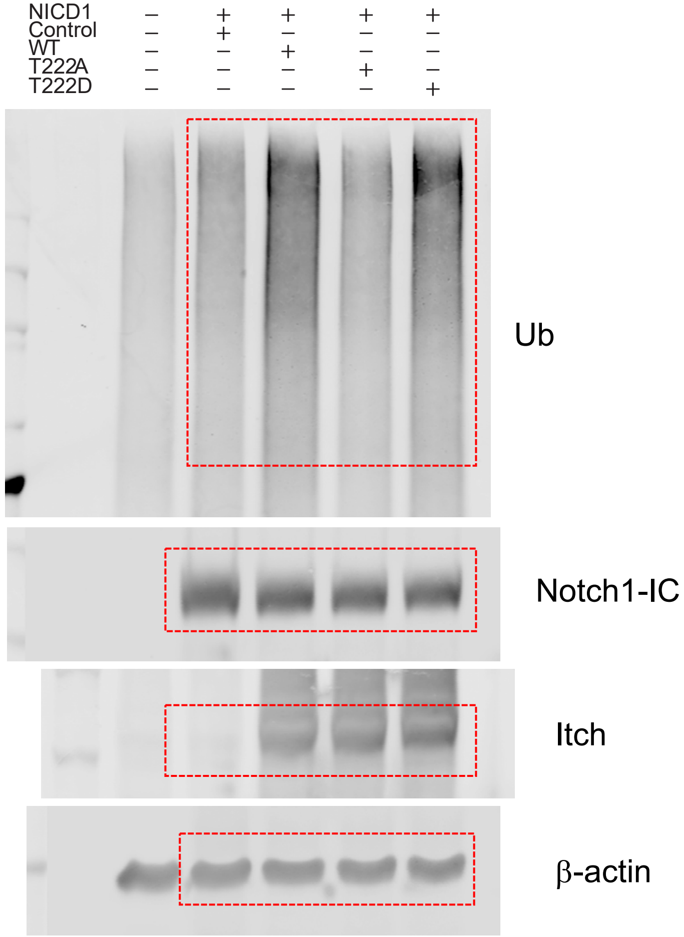

Fig. S1A

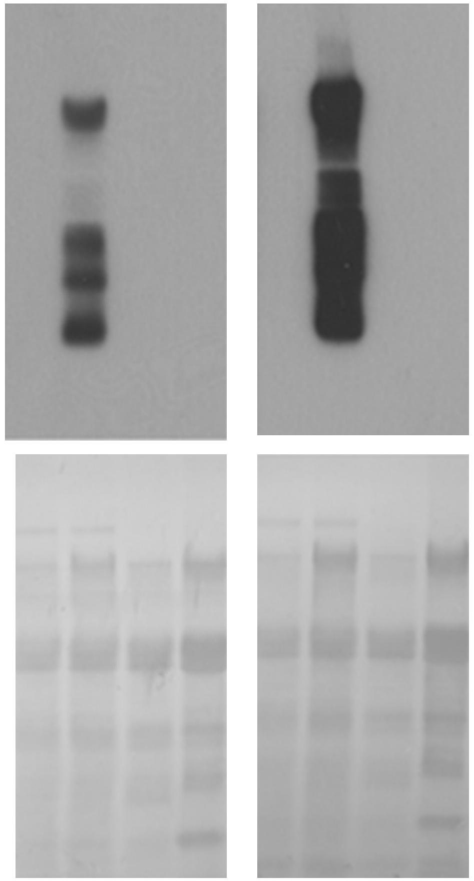

pJIP1@S235 pJIP1@T205

Fig. S1B

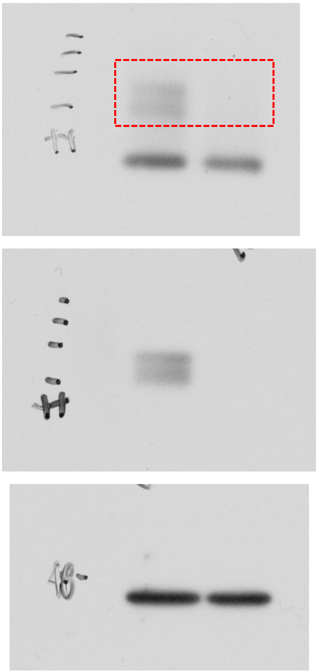

Fig. S1C

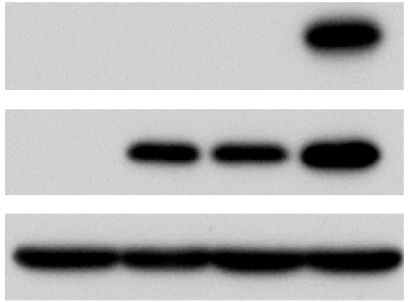

Fig. S1D

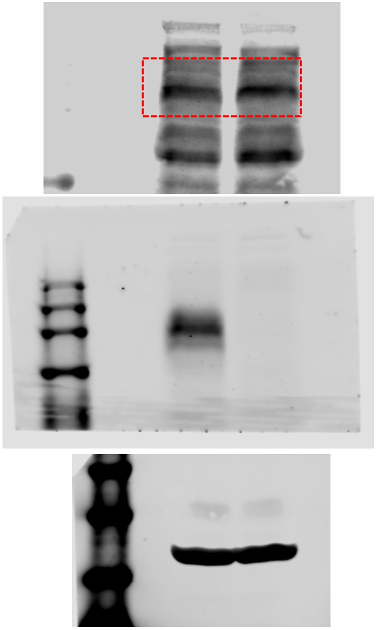

Fig. S2A

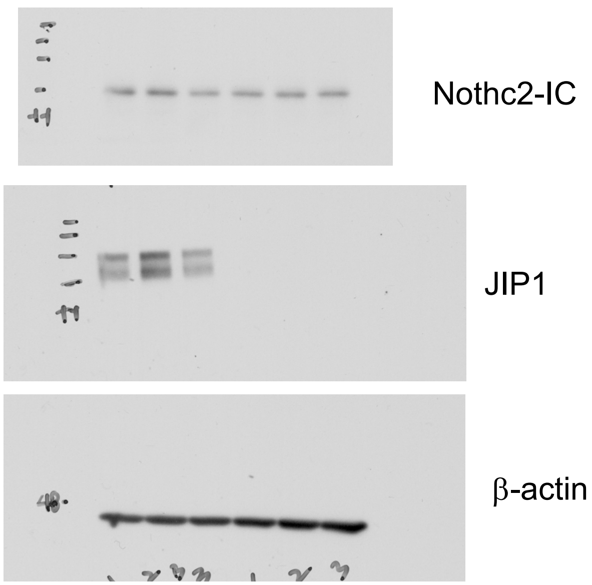

Fig. S2B

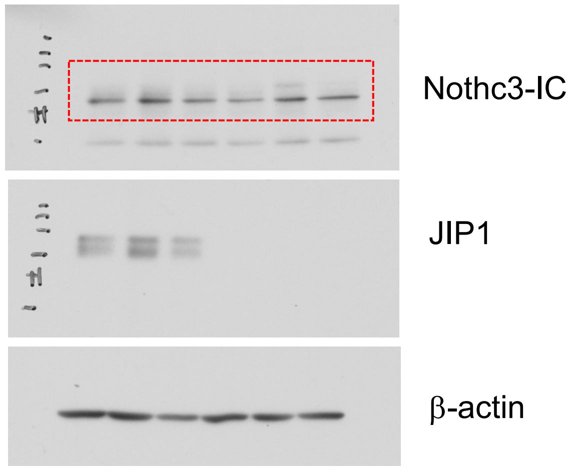

Fig. S3A

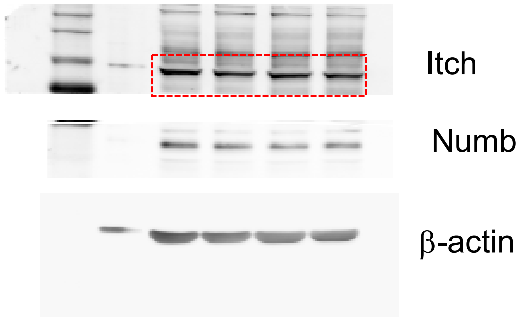

Fig. S3D

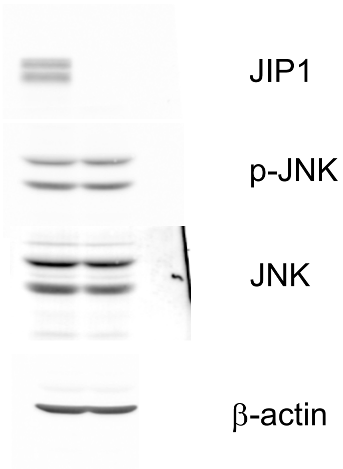

Fig. S3E

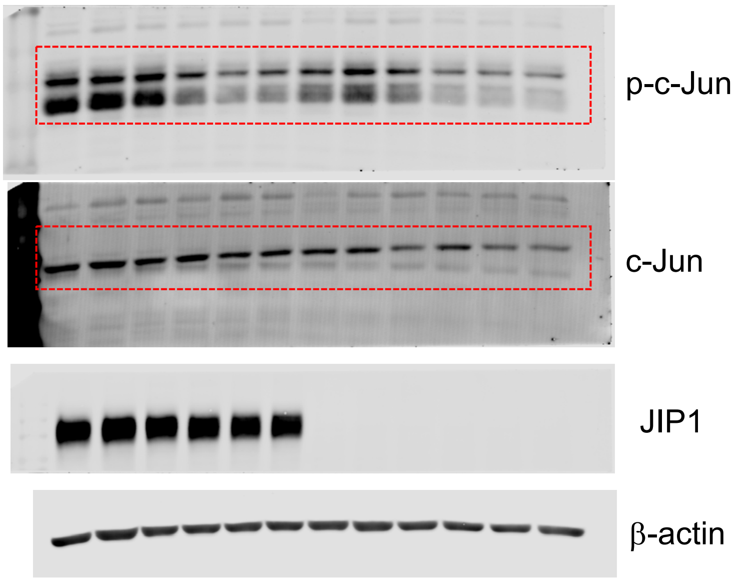

Fig. S4

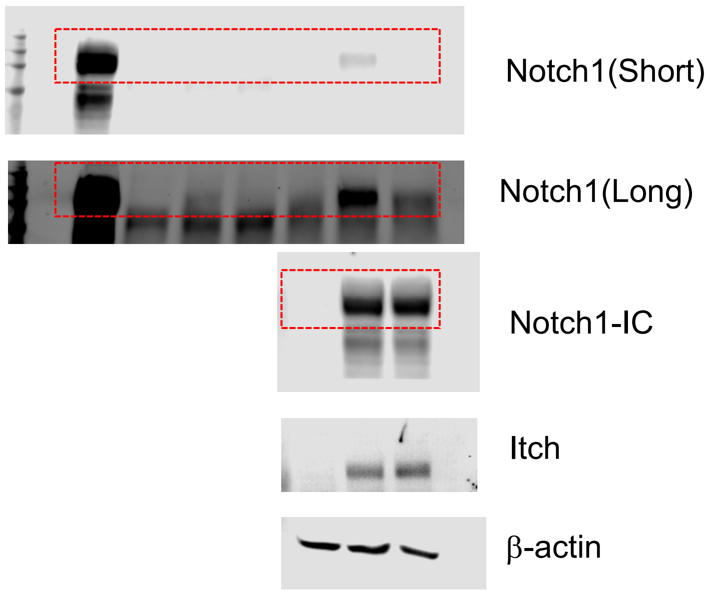

Supplement: Supplementary file 3 — Additional file 3. Western blots. [file 12915_2022_1312_MOESM3_ESM.pdf]
